# Heteroleptic Copper(I) Complexes of “Scorpionate” Bis-pyrazolyl Carboxylate Ligand with Auxiliary Phosphine as Potential Anticancer Agents: An Insight into Cytotoxic Mode

**DOI:** 10.1038/srep45229

**Published:** 2017-03-24

**Authors:** Rais Ahmad Khan, Mohammad Usman, Rajakumar Dhivya, Perumalsamy Balaji, Ali Alsalme, Hamad AlLohedan, Farukh Arjmand, Khalid AlFarhan, Mohammad Abdulkader Akbarsha, Fabio Marchetti, Claudio Pettinari, Sartaj Tabassum

**Affiliations:** 1 Department of Chemistry, College of Science, King Saud University, Riyadh 11451, Saudi Arabia; 2 Department of Chemistry, Aligarh Muslim University, Aligarh-202002, India; 3 National Center for Alternatives for Animal Experiments, Bharathidasan University, Tiruchirappalli, 620024, India; 4 Surfactant Research Chair, College of Science, King Saud University, Riyadh 11451, Saudi Arabia; 5 Dipartimento di Scienze Chimiche, Universita´ degli Studi di Camerino, via S. Agostino 1,62032 Camerino, Macerata, Italy

## Abstract

New copper(I) complexes [CuCl(PPh_3_)(L)] (**1:** L = L_A_ = 4-carboxyphenyl)bis(3,5-dimethylpyrazolyl)methane; (**2:** L = L_B_ = 3-carboxyphenyl)bis(3,5-dimethylpyrazolyl)methane) were prepared and characterised by elemental analysis and various spectroscopic techniques such as FT-IR, NMR, UV–Vis, and ESI-MS. The molecular structures of complexes **1** and **2** were analyzed by theoretical B3LYP/DFT method. Furthermore, *in vitro* DNA binding studies were carried out to check the ability of complexes **1** and **2** to interact with native calf thymus DNA (CT-DNA) using absorption titration, fluorescence quenching and circular dichroism, which is indicative of more avid binding of the complex **1**. Moreover, DNA mobility assay was also conducted to study the concentration-dependent cleavage pattern of pBR322 DNA by complex **1**, and the role of ROS species to have a mechanistic insight on the cleavage pattern, which ascertained substantial roles by both hydrolytic and oxidative pathways. Additionally, we analyzed the potential of the interaction of complex **1** with DNA and enzyme (Topo I and II) with the aid of molecular modeling. Furthermore, cytotoxic activity of complex **1** was tested against HepG2 cancer cell lines. Thus, the potential of the complex **1** is promising though further *in vivo* investigations may be required before subjecting it to clinical trials.

Cancer is ranked the second most common cause of death, only after cardiovascular diseases. Hepatocellular carcinoma or hepatic/liver cancer is the sixth most widespread cancer and the third leading cause of cancer-associated deaths^1^. In the year 2016, in the USA alone, new cases and deaths due to liver/intrahepatic bile duct cancer were found to be 39230 (incidence) and 27170 (mortality)^2^. Several research efforts have been made to deal with liver cancer, which include the area of metal-based drugs for cancer chemotherapy. The field of metallodrugs came to be recognized after the foundation laid by the serendipitous discovery of cisplatin (cis-diamminedichloroplatinum(II)). Cisplatin exhibited wide applications as a chemotherapeutic agent, but it has also been found to produce severe side effects^3^,^4^,^5^,^6^,^7^,^8^,^9^. Since then there has been a hectic search for better metal-based cancer chemotherapeutic drugs. The unique properties of metal ions can be taken to advantage in the designing of new potential anticancer drugs with different mechanisms of action for targeting various cancer cells^10^,^11^,^12^,^13^. Various metal complexes capable of binding efficiently to and cleave DNA under physiological environment are regarded as potential chemotherapeutic agents^14^,^15^,^16^,^17^,^18^,^19^,^20^,^21^. In this regard, copper has attracted significant interest, since it plays key roles in biochemical processes such as nitrite reductase^22^, amine oxidases^23^, superoxide dismutase^24^, catechol oxidase^25^ and tyrosinase^26^. Copper(II) complexes have been intensily studied^10^,^27^ whereas the potential of Cu(I) complexes has been scarcely tested as antimicrobial^28^, antiviral^29^, antifungal^30^ and anticancer drugs^17^,^31^,^32^,^33^,^34^. Mostly, coordination compounds having S-/N-donor and/or phosphine ligands are highly suitable candidates for clinical investigation, given their proven cytotoxicity on cancer cells and high stability in aqueous solution^35^. Most likely, Cu(II) complexes exhibit enzyme inhibition properties^36^, which lead to the death of the cancer cells. Copper(I) compounds mainly containing N/S-donor ligands have been reported during the past several years^10^,^27^,^37^. In fact, due to the binding properties of pyrazoles, triazoles, imidazoles, oxazoles, thiadiazoles and thiazoles towards biomolecules makes the Cu(I) complexes, promising entities for pharmaceutical applications as chemotherapeutic agents. Copper(I) complexes have also been reported to act as enzyme inhibitors, particularly the topoisomerases, topo I and topo II, and multi-protein proteasomes which lead to death of cancer cells^38^,^39^.

The first phosphine metal complex in clinical use was auranofin (a tetraacetylthioglucose derivative of triethylphosphine gold(I)) introduced to treat rheumatoid arthritis^40^,^41^. Numerous studies revealed the potential of auranofin to act as potent cytotoxic agent *in vitro* and *in vivo*^42^,^43^. These findings prompted several investigations to focus on the anticancer properties and other biological activities of various metal phosphine complexes. Bis(pyrazolyl)alkanes are a class of ligands endowed with N, N copper-coordinating abilities and, thus, provide flattened tetrahedral metal geometry. A series of Cu(I) mixed-complexes containing sodium bis(1,2,4-triazol-1-yl)acetate or sodium bis(3,5-dimethyl-pyrazol-1-yl)acetate ligands, and phosphine co-ligands were tested for their cytotoxic potential against a panel of human carcinoma cell lines^10^,^27^,^44^. In the light of our group’s experience in this field and the particular interest for scorpionate ligands and their complexes^45^,^46^,^47^, we have herein synthesized two ligands 4-carboxyphenyl)bis(3,5-dimethylpyrazolyl)methane (L_A_) and 3-carboxyphenyl) –bis(3,5-dimethylpyrazolyl)methane (L_B_) reported earlier by Carrano *et al*.^48^,^49^.

Thus, we have designed and synthesized two new copper(I) complexes **1** and **2** by interaction of bis(3,5-dimethylpyrazolyl)-derived scorpionate ligands L_A_ and L_B_ with the precursor CuCl in the presence of triphenylphosphine (PPh_3_). **1** and **2** were characterized using various spectroscopic tools, elemental analysis, and TD-DFT analysis. Preliminary studies of binding complexes **1** and **2** with CT-DNA were carried out adopting UV-visible, fluorescence, and circular dichroism techniques. Later, DNA cleavage experiments were carried out to have a mechanistic insight into the binding mode of the complexes **1** with DNA. Subsequently, the complex **1** was tested against HepG2 human hepatocarcinoma cell to find its cytotoxic property.

## Results and Discussion

### Synthesis and Characterization

**Figure 1:**
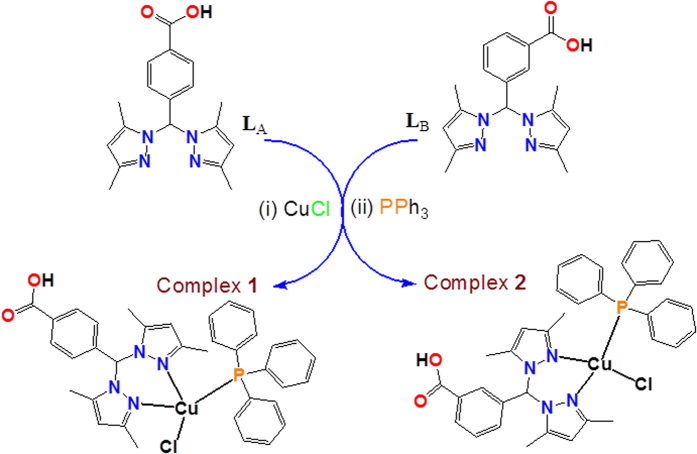
Schematic representation of the synthesis of **1** and **2**.

The mixed-ligand copper(I) complexes were prepared by reacting CuCl in acetonitrile or methanol/acetonitrile solutions with the ligands L_A_ or L_B_ and triphenylphosphine, in the equimolar stoichiometric ratio at room temperature (see Fig. 1). Both the complexes **1** and **2** were isolated in good yields of ~60% as white crystalline powders. Despite several attempts, we failed to isolate single crystals suitable for X-ray diffraction. However, the positive electrospray mass spectra (ESI MS, +ve), FT-IR and NMR spectra were in close agreement by the proposed structures (see experimental section and for more detail see Figures S1–S8 in Supplementary Information). The purity of the complexes was confirmed by elemental analyses. They are stable in air as well as in solution phases for 72 h.

The FTIR spectra of the complexes **1** and **2** showed the presence of all characteristic peaks. The intense signal around 1715 cm^−1^ for **1** and 1706 cm^−1^ for **2** marks the presence of carboxylate moiety associated with asymmetric COO^−^ modes. The spectra showed shift in the C = N aromatic peaks of the pyrazolyl ring of the ligands and appears at around 1559 and 1558 cm^−1^ for **1** and **2**, respectively, which ascertain the coordination of the nitrogen to the Cu centre. Furthermore the sharp peak at 1093 cm^−1^ marks the presence of coordinated PPh_3_ auxiliary ligand in both the complexes **1** and **2.**

Both complexes were characterized by ^1^HNMR and ^31^P NMR in CD_3_OD-d_4_ solvent. ^1^HNMR of the complexes **1** and **2** exhibited signals for aliphatic as well as aromatic protons in accordance with the proposed structures. The resonances for the aromatic rings of the ligand were observed in the range 8.04–6.24 ppm. The shifts in the signals 8.01, 7.45, 6.62 ppm for 1 and 8.04, 7.61, 7.42, and 6.82 ppm for 2, are ascribed to the aromatic benzene ring of the ligand. The peak for pyrazolyl CH appeared at 6.24 and 6.27 ppm, for **1** and **2**, respectively. The resonances of the methyl groups of pyrazolyl moieties exhibited in the range of 2.61–2.60 and 2.13–2.06 as singlets in both complexes. ^31^P NMR of the complexes **1** and **2** gave rise to signals at +3.96 and +4.58 ppm which confirms the PPh_3_ is coordination with the metal centre. ESI-MS (positive) was done in the solution which gave the peak at 649.3 assigned to [C_36_H_35_CuN_4_O_2_P]^+^- 1 H^+^ at 100% relative intensity. Thus, all characterization studies corroborate well with proposed structure of the complexes **1** and **2**.

To get an insight into the electronic structure of the complexes, we carried out B3LYP/DFT studies.

### Computational Chemistry

#### The Ground State Structures by DFT Calculation

**Figure 2:**
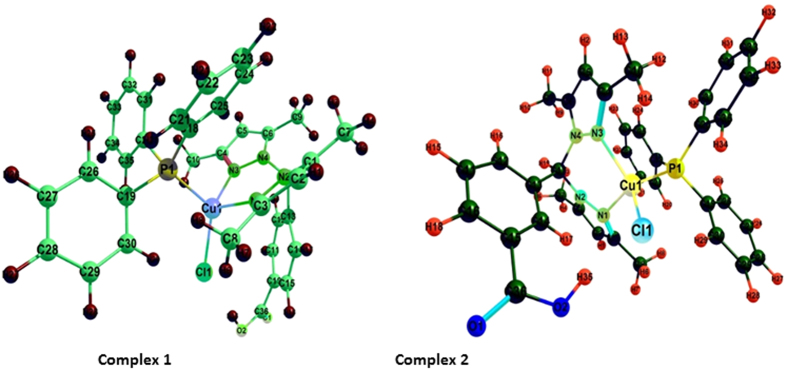
Gas phase B3LYP/DFT optimized structure of complexes **1** and **2**.

**Figure 3:**
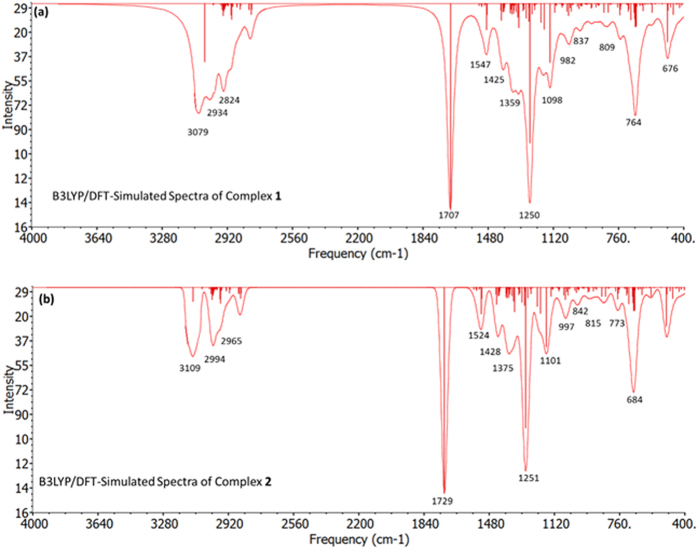
B3LYP/DFT simulated vibrational spectra of (**a**) complex **1** and (**b**) complex **2**.

**Figure 4:**
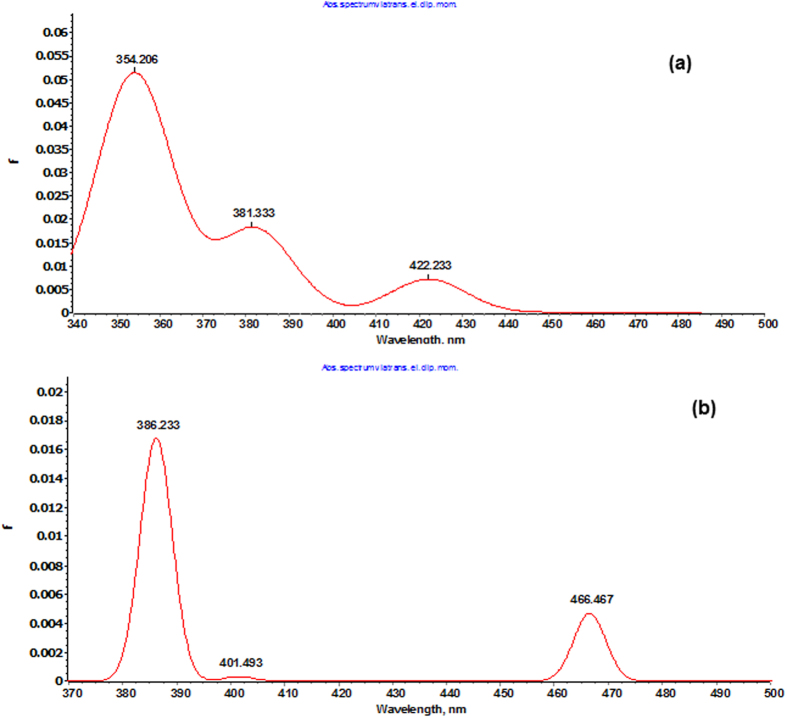
B3LYP/TDDFT simulated electronic absorption spectra of complexes **1** and **2.**

Density functional theory (DFT) calculations were accomplished to investigate the electronic structure and geometrical parameters, as the crystal structure of complexes **1** and **2** have not been obtained. The optimized structures of complexes **1** and **2** at ground state are depicted in Fig. 2. The geometrical parameters *viz.* calculated bond lengths, bond angles and atomic coordinates of the optimized structure of complexes **1** and **2** are given in Tables S1–S4. The calculated geometrical parameters are in rational agreement with the previously reported single crystal X-ray data in different papers^48^,^49^. The vibrational and electronic absorption spectra have also been simulated to validate the proposed structure of complexes **1** and **2** (Figs 3 and 4). The calculated frequencies were found within the range, as shown in Table 1. Slight variation has been observed in simulated vibrational spectra because experimental values contained both harmonic and anharmonic vibrational frequencies while the calculated values depicted only harmonic vibrations, but the pattern of spectra was quite similar in both the cases which validate the proposed structure.

**Figure 5:**
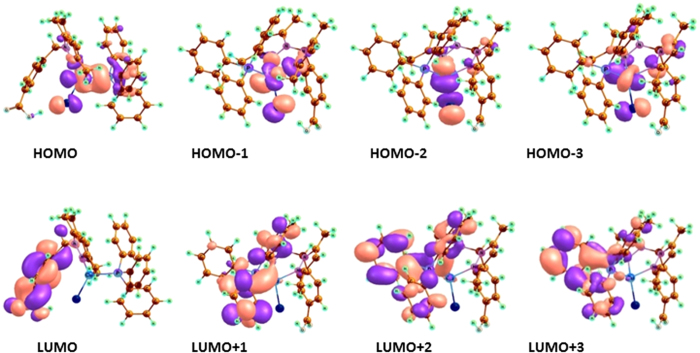
Frontier MOs contour plots (isovalue 0.03) of complex **1** using the B3LYP/DFT method.

**Figure 6:**
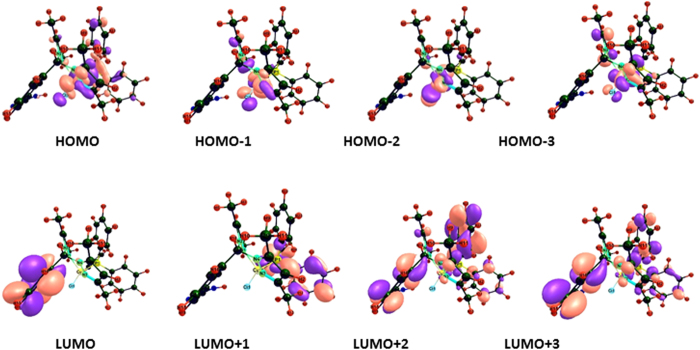
Frontier MOs contour plots (isovalue 0.03) of complex **2** using the B3LYP/DFT method.

The structure of complexes **1** and **2** in the gas phase was determined to be metal centered complexes with distorted tetrahedral geometry, in which the 3d^10^ copper (I) center is surrounded by two nitrogen atoms of the ligand, one phosphorous atom of PPh_3_ and one chlorine atom. An exhaustive analysis of the highest occupied molecular orbitals (HOMOs) and lowest unoccupied molecular orbitals (LUMOs) of the Cu(I) based complexes is listed in Table S5. In the complexes **1** and **2**, there are five HOMOs (HOMO to HOMO – 4) predominantly 3d orbitals of the copper (I) ion, but significant contributions from the phosphine ligands also are evident. The LUMO is almost entirely made up of atomic orbitals of the scorpionate ligand while LUMO−1 to LUMO-3 from the PPh_3_ moiety, as shown in Figs 5 and 6.

Furthermore, the B3LYP/TDDFT calculations were performed on complexes **1** and **2** to simulate the UV-Vis spectra to validate the tentative spectral assignment. The absorption bands exhibited by MLCT transitions are in the range of 400–600 nm, that is excitation from the highest occupied Cu 3d orbitals to the lowest unoccupied scorpionate ligand π* orbitals. This peak relates to the lowest-energy excitation, which displays strong HOMO to LUMO character. The simulated spectra shifted towards the longer wavelength which could be due to the basis set discrepancies.

### DNA Binding Studies

#### Uv-Vis Absorption Titration

**Figure 7:**
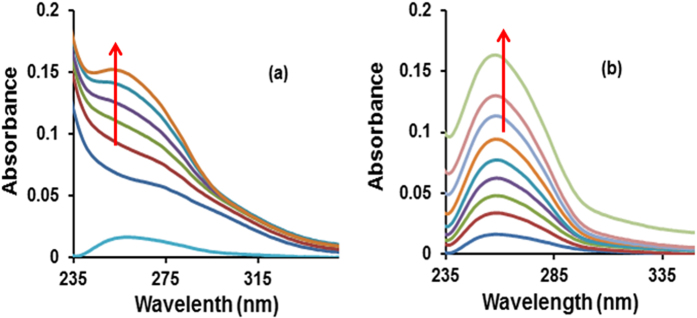
Absorption spectral traces of complexes (**a**) **1** and (**b**) **2** in 95:5% H_2_O:DMSO/5 mM Tris HCl-50 mM NaCl buffer at pH 7.4 upon addition of CT DNA.

Electronic absorption spectroscopy has been effectively used for decades to study the mode and extent of interaction of potential drug molecules with DNA^50^,^51^,^52^,^53^. The spectral behavior of **1** and **2** (in 95:5, H_2_O: DMSO solution) was studied in the absence and presence of CT-DNA (in 5 mM Tris-HCl/50 mM NaCl buffer, pH 7.4). The spectra are depicted in Fig. 7a,b.

Upon gradual addition of CT-DNA to the solutions of complexes **1** and **2,** a “hyperchromism” was observed, with an evident red-shift of 2–4 nm. This suggests an electrostatic mode of interaction with groove binding affinity. The binding strengths of **1** and **2** were quantified from the values of intrinsic binding constant K_b_, determined by using Wolfe–Shimer equation^54^. The K_b_ values were found to be 4.21 × 10^4^ M^−1^ and 1.37 × 10^4^ for **1** and **2**, respectively.

#### Ethidium Bromide Displacement Assay

**Figure 8:**
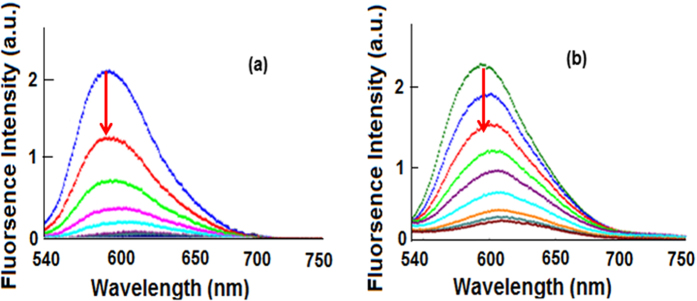
Emission spectra of EthBr-CTDNA in the absence and presence of compound (**a**) **1** and (**b**) **2** in 5 mMTris–HCl/50 mM NaCl buffer at pH 7.4. Arrows show the emission intensity changes upon increasing the concentration of the complexes.

We carried out competitive binding studies by using ethidium bromide (EthBr). This assay differentiates between intercalator and non-intercalator^50^,^51^. The DNA-EthBr (λ_em_ = 590 nm) emission spectrum was taken as control. As such, EthBr alone is weakly emissive, but when it is bound to DNA, it becomes highly emissive. Binding of EthBr with DNA in a stoichiometric ratio and then treating it with the tested molecule give rise to a change in fluorescence intensity. A significant decrease in emission intensity of DNA-EthBr suggests a displacement of EthBr molecule, which is the characteristic feature of an intercalator. The emission spectra of EthBr-DNA in the absence and presence of **1** and **2** are shown in Fig. 8a,b. The spectra clearly reveal intercalating ability of both **1** and **2**, by depleting EthBr from the bound EthBr-DNA and quenching the fluorescence intensity significantly. Despite the fact that our copper(I) complexes do not have a perfect planar geometry, the aromatic rings of the phosphine moiety seem to play a significant role in the intercalation. In fact, the extent of quenching is quite substantial. However, non-intercalative interactions cannot be ruled out completely.

The quenching strength of the complexes **1** and **2** for EthBr-DNA was calculated by using the Stern- Volmer equation.


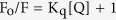


where “F_o_” and “F” are the fluorescence intensities in the absence and presence of the complex, respectively. “K_q_” is the quenching constant and [Q] is the concentration of the complex. The slope of the ratio F_o_/F vs. [Q] plot gives the value of “K_q_”. The K_q_ values obtained for complexes **1** and **2** are 8.7 × 10^5^ and 4.8 × 10^5^ M^−1^, respectively. Moreover, the apparent binding constants (K_app_) were calculated using the Eqn. below:


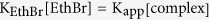


where “K_EthBr_” (1.0 × 10^7^ M^−1^) is the binding constant of EthBr with DNA, [EB] is the concentration of EthBr (10 μM) and “[complex]” is the concentration of the complex at which the intensity of EthBr-DNA is reduced. The values of K_app_ were found to be 10.0 × 10^5^ M^−1^ and 3.3 × 10^5^ M^−1^ for **1** and **2**, respectively. These findings are in agreement with the results and conclusions of the absorption spectral studies.

#### Circular Dichroism

Since circular dichroism (CD) is a technique highly sensitive to changes in the conformation of the chiral structure of DNA^52^,^55^, it can be used to study the subtle variations upon its interaction with small molecules^56^,^57^,^58^,^59^. The CD spectrum of CT-DNA exhibits a positive band at 275 nm (UV, λ_max_ = 260 nm) caused by base stacking between the compounds and DNA, and a negative band at 245 nm caused by helicity, which is characteristic of DNA in right-handed B form. Intercalation enhances the intensity of both positive and negative bands, whereas groove binding and electrostatic interactions of complexes cause less or no perturbation on the base stacking and helicity bands.

**Figure 9:**
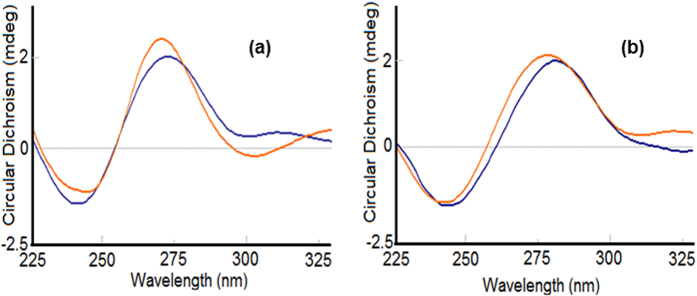
Circular dichroism spectra of CT DNA in the absence and presence of compound (**a**) **1** and, (**b**) **2** in 5 mM Tris–HCl/50 mM NaCl buffer at pH 7.4.

The spectrum of CT-DNA with complex **2** displayed a slight change in the conformation, which can be attributed to groove binding affinity along with electrostatic interactions (Fig. 9a) whereas, for complex **1** the CD spectrum displayed an increase in ellipticity on both ends, i.e., 275 nm as well as 245 nm, which is indicative of intercalation. Therefore, the complex **1** is a better potential candidate to penetrate into the helical structure of the DNA, leading to a significant perturbation in the DNA structure (Fig. 9b). However, the extent of intercalation is not very great, as still the conical B-form of the DNA is maintained, and thus a partial intercalative mode of action of **1** with DNA can be proposed.

#### Gel Mobility Assay

**Figure 10:**
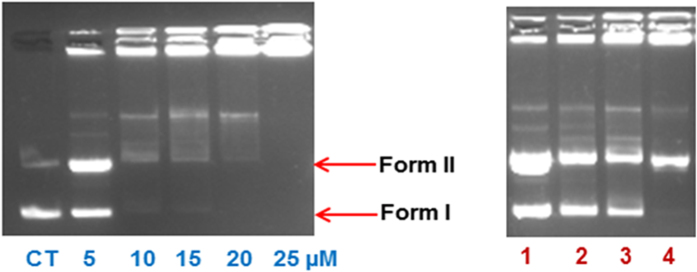
(**a)** Electrophoretic pattern of pBR322 DNA (100 ng) with increasing concentration of complex **1** (5–25 μM) after 30 min incubation time in buffer (5 mMTris-HCl/50 mM NaCl, pH = 7.2 at 25 °C) (concentration dependent). **(b)** Cleavage pattern of pBR322 plasmid DNA (100 ng) by complex **1** (10 μM) in the presence of reactive oxygen species viz., DMSO (0.4 mM), EtOH (0.4 mM), NaN_3_ (0.4 mM) and SOD (10 U), after incubation for 30 min in the buffer (5 mM Tris-HCl/50 mM NaCl, pH = 7.2 at 25 °C.

The DNA cleavage activity of complex **1** was analyzed by monitoring the electrophoretic pattern of pBR322 DNA alone and upon treatment with increasing concentrations of the complex (5–25 μM) (see Fig. 10a). The changes observed in the electrophoretic mobility pattern of pBR322 DNA, as a result of interactions with **1**, are quite evident. The appearance of new bands is attributed to DNA-complex interaction. The fast migrating band is the form I (supercoiled DNA), evident as a prominent band in control, whereas the slow moving band is form II (nicked form of DNA)^59^,^60^. In such type of complexes, coordinative type of bonding between the metal centre and DNA is less likely because of the favorable Cu(I) (soft acid) and soft donor ligand viz, -P and –S, and this coordination requires binding with N-donor (hard donor ligand)^61^. Intercalation and/or inter-strand cross-linking cannot be totally excluded, due to the presence of the band located around the well of the gel. This band can be marked as the complex-DNA adduct formation, which has increased the molecular weight, and the adduct is unable to take part in electrophoretic mobility as compared to the control. However, the mobility pattern revealed the concentration-dependent cleavage activity of the complex.

To probe into the potential mechanism, we investigated the effect of reactive oxygen species (ROS) on the cleavage pattern of pBR322 DNA, upon interaction with complex **1** (see Fig. 10b)^62^,^63^. Thus, we treated pBR322 DNA and complex **1** (10 μM) with hydroxyl radical scavengers (lane 1, DMSO and lane 2, EtOH), singlet oxygen quencher (NaN_3_, lane 3) and superoxide oxygen scavenger (SOD, lane 4), under identical conditions as above. In all the cases (lanes1–4), the inhibitory effect is significantly evident, which means the presence of all the ROS (^•^OH, ^1^O_2_, O_2_^–^) as responsible for the cleavage, but it does not diminish the effect of inter-cross linking and/or intercalative effect on cleavage of DNA. Thus, hydrolytic as well as oxidative mechanistic pathways can play a significant role in the cleavage pattern.

**Figure 11:**
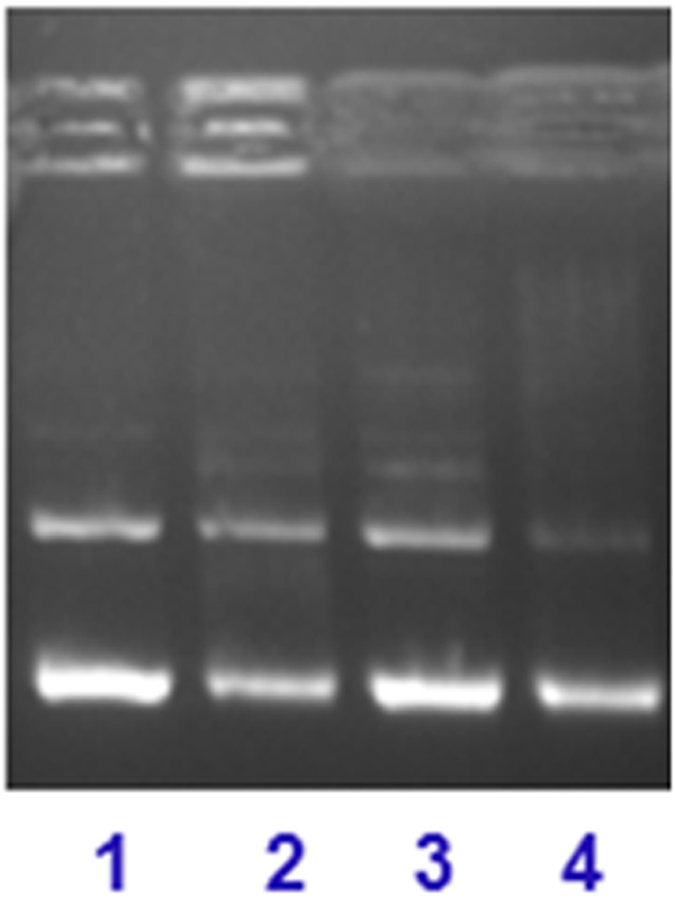
Gel mobility pattern for the cleavage of pBR322 DNA (100 ng) by complex **1** (10 μM) in the presence of major/minor groove recognition element: lane 1, **1** + Distamycin + DNA; lane 2, **1** + Methyl green + DNA; Lane 3, **1** + DAPI + DNA; lane 4, DNA only, control, at 25 °C after incubation for 30 min.

To obtain further insight into the binding site of the complex **1** with DNA, we studied the mobility in the presence of recognition elements such as methyl green (major groove binder) and distamycin, 4′,6-Diamidino-2-phenylindole “DAPI” (minor groove binder)^63^,^64^,^65^. The pattern exhibited higher affinity towards the minor grooves of DNA, as in this case the cleavage is significantly inhibited (lanes 1 and 3), whereas in the presence of the major groove binder the cleavage is not entirely inhibited (lane 2) (Fig. 11). This further strengthens our hypothesis of intercalative and/or inter-cross linking property of complex **1** with DNA.

### Molecular Modeling Studies

#### Molecular docking with DNA

**Figure 12:**
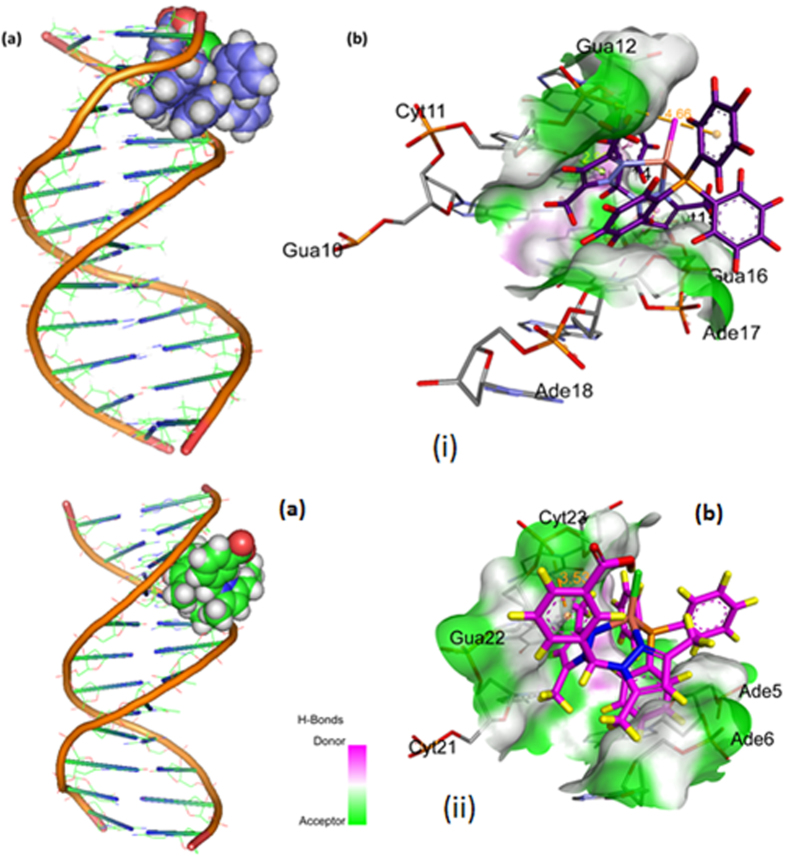
Molecular docked model of **(i)** complex **1** and **(ii)** complex **2,** in the **(a)** cavity of minor groove of DNA **(b)** binding site interactions with hydrogen bonding donor (purple) and acceptor (green) surface of minor groove residues.

The docked model (Fig. 12) revealed that complexes **1** and **2** were tailored tightly into the curved contour of the targeted DNA minor groove within G–C rich region, and scorpionate ligand moiety slightly twisted the interior hydrophobic surface of DNA in such a way that planar part of the aromatic rings made favourable stacking interactions between DNA base pairs and produced Van der Waals interaction and hydrophobic contacts with the DNA functional groups which would define the stability of groove, detailed description of which is given in Table 2.

The resulting relative total energy of docked structures were found to be −259.55 and −247.68 eV, indicating the greater binding affinity between DNA and complex **1** as compare to complex **2**, correlating well with the experimental DNA binding studies and minor groove binder using DAPI assay for **1**.

#### Molecular docking with Human–DNA–Topo–I

**Figure 13:**
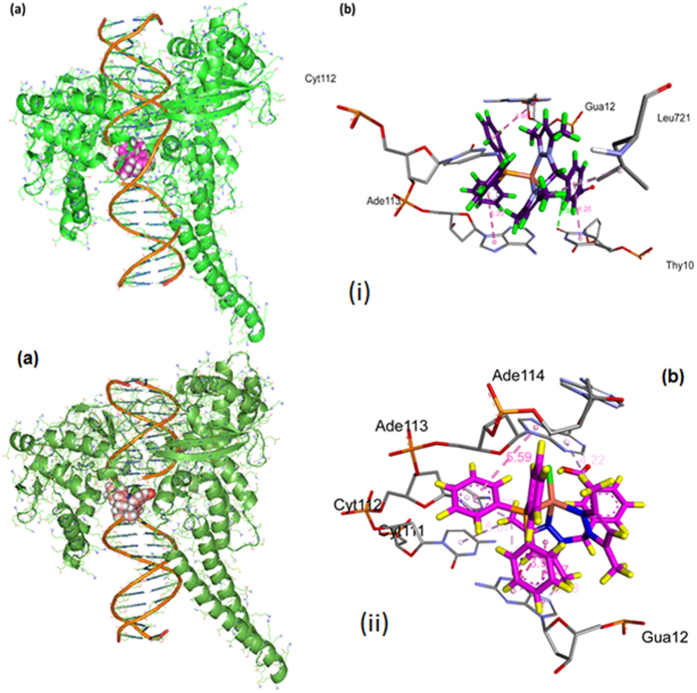
Molecular docked model of **(i)** complex **1** and **(ii)** complex **2,** (**a**) with human-DNA-Topo-I (70 kDa) (PDB ID: 1SC7) (**b**) Non-covalent interaction of complex **1** with the active site residues.

In order to determine the mechanistic basis for the inhibitory action and to obtain accurate binding mode of the hits on Topo–I, the complexes **1** and **2** were studied. The resulting docked models exhibited a dual mode of binding on Topo–I due to conformation changes (viz, structural flexibility) of the interacting scorpionate moiety as well as anionic copper(I) coordinates chlorine atom. Figure 13 shows aromatic rings of complex **1** approaching the DNA cleavage site in the Topo–I–DNA complex and forming a stable complex through π–π stacking interactions between the purine ring of G11 (+1) and pyrimidine ring of T10 (−1) in the minor groove on the scissile strand and C112 and A113 on the non–scissile strand, parallel to the plane of base pairs.

The carboxylic hydrogen atom of the complex **1** was H–bonded to Leu 721, which is considered an essential amino acid that interacts with the ligand in the DNA–Topo–I active site, whereas aromatic rings facing perpendicularly to the plane of base pairs which strongly block the rewinding step of the phosphoester. Furthermore, DNA-intercalating forces were much more important than hydrogen bonding of the ligand to the surrounding amino acid residues of the protein, or to the base pairs. Our molecular docking study proved the importance of DNA intercalating ability of **1** and **2** as well as H–bond and π–π stacking hydrophobic interaction with the enzyme in the cleavage site viz, non-covalent interaction listed in Table 3. This result suggests that blocking the religation of the G11 hydroxyl group could be the main design point for novel Topo–I inhibitors. The resulting binding energy of minimum energy-docked structure was found to be −305.65 and −292.16 eV, respectively, revealing the potent greater binding affinity between Human–DNA–Topo–I and complex **1** as compared to complex **2**, The model studies are suggestive of potential basis for conceivable design of novel anticancer drugs targeting active site of Topo–I.

#### Molecular docking with Human–DNA–Topo–II

**Figure 14:**
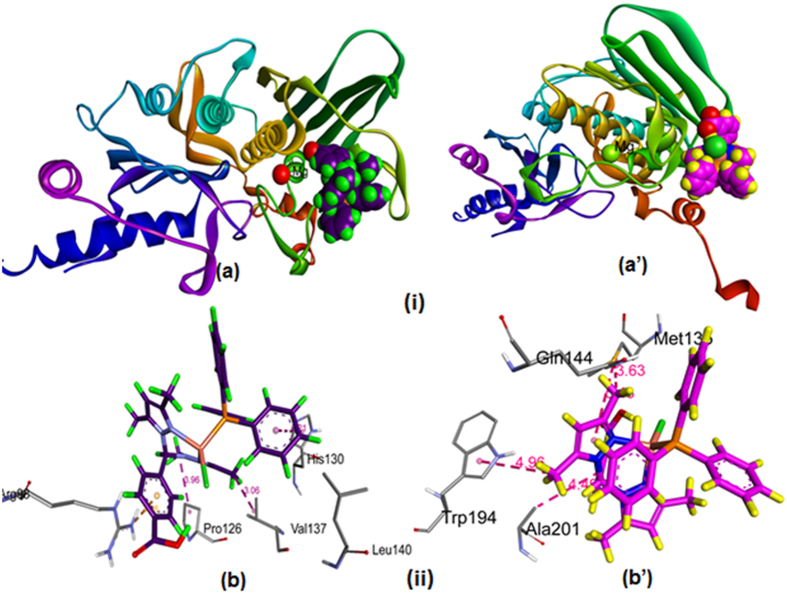
Molecular docked model of **(i) (a)** complex **1** and **(a’)** complex **2** into the ATP binding pocket of human Topo-IIα (PDB ID: 1ZXM).**(ii) (b)** complex **1** and **(b’)** complex **2**, the non-covalent interaction with the Topo IIα.

To study the molecular basis of interaction and to rationalize the observed enzymatic activity, molecular docking studies of complexes **1** and **2** with the ATP-binding domain of human Topo-IIα (PDB ID: 1ZXM) were carried out. The docking models showed that complexes **1** and **2** were approaching the middle of ATP-binding pocket and stabilized by strong hydrophobic interactions with His130, Pro126, Val137, Arg98, Leu140 and Met135, Trp194, and Ala201 for **1** and **2**, respectively (Fig. 14 and Table 4). These multiple interactions between complexes and the residues in the ATPase domain and the magnesium ion suggest that the metal complexes can form a strong binding interaction with Topo II, preventing the entry of ATP. The resulting binding energy of docked complexes **1** and **2** with DNA binding site of topoisomerase II were found to be −273.01 and −176.35 eV, indicating greater affinity of complex **1** towards the ATP pocket.

### *In Vitro* Anticancer Studies

#### MTT assay

The cytotoxic effect of complexes **1** and **2** was examined on cultured HepG2 liver carcinoma cells by exposing cells for 24 h to the medium containing the complexes each at 1–10 μM concentrations. The complexes inhibited the growth of the liver cancer cells in a dose-dependent manner. The results were determined according to the dose values of exposure of the complex required to reduce survival of the cells to 50% (IC_50_). The IC_50_ values are given in Table 5. The complex **1** showed effective cytotoxic activity against the liver cancer cell compared to the standard drug, Cisplatin.

#### Hoechst staining

**Figure 15:**
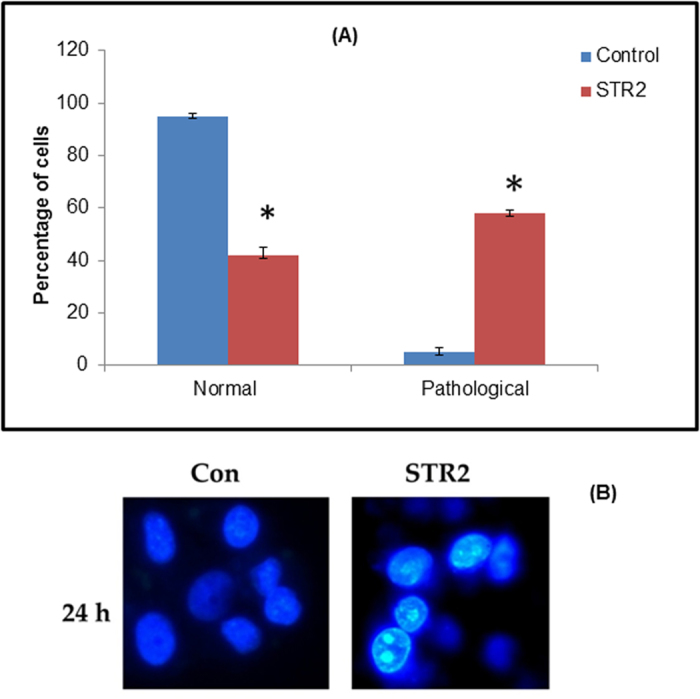
(**A**) Hoechst 33258 staining of the complex **1**-induced apoptosis of HepG2 cells. The graph shows the manual count of apoptotic cells as a percentage (data are mean% ± SD% of three experiments). (**B**) Representative morphological changes observed in HepG2 cells treated with **1** (STR2) adopting Hoechst 33258 staining.

After treatment with the IC_50_ concentration of the complex **1** for 24 h, the cells were observed for cytological changes adopting Hoechst 33258 staining. The observations revealed that the complex **1** brought about marginalization and/or fragmentation of chromatin, bi-nucleation, cytoplasmic vacuolation, nuclear swelling, membrane blebbing and late apoptosis indication of dot-like chromatin and condensation in the HepG2 cells (Fig. 15). These cytological changes indicate that the cells were committed to a specific mode of cell death, either apoptosis or necrosis.

#### Acridine Orange/Ethidium Bromide Staining

**Figure 16:**
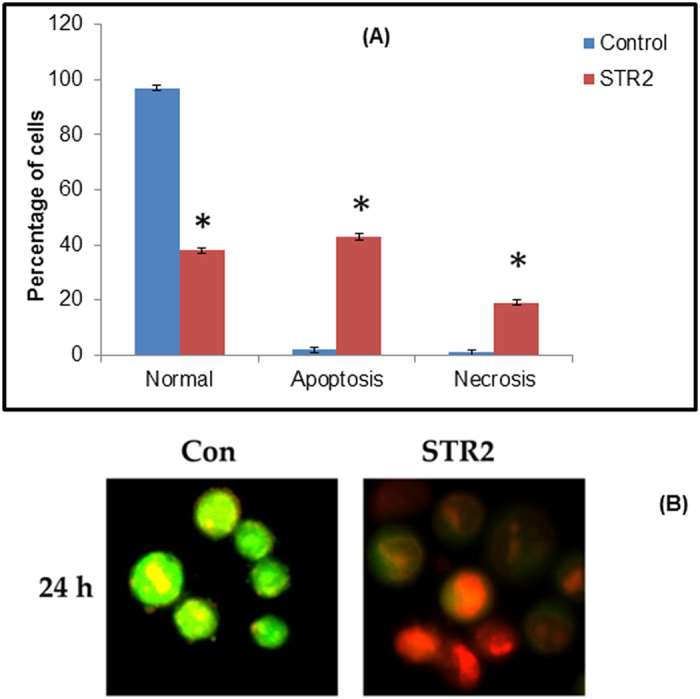
AO/EB staining of the complex **1**-induced apoptosis of HepG2 cells. The graph shows the manual count of apoptotic cells as a percentage (data are mean% ± SD% of three experiments). (**B**) Representative morphological changes observed in HepG2 cells treated with **1** by dopting AO/EB staining.

To confirm the mode of cell death, we adopted acridine orange and ethidium bromide (AO and EB) staining. Observation of AO-EB stained cells in a fluorescent microscope revealed apoptosis greatly, but also necrosis to a certain extent, from the perspective of fluorescence emission. After HepG2 cells had been exposed to IC_50_ concentration of the complex **1** for 24 h, the cells were assigned to four types, according to the fluorescence emission and the morphological features of chromatin condensation in the stained nuclei (Fig. 16); (i) viable cells had uniformly green fluorescing nuclei with a highly organized structure; (ii) early apoptotic cells had green fluorescing nuclei, but the perinuclear chromatin condensation was visible as bright green patches or fragments; (iii) late apoptotic cells had orange–red fluorescing nuclei with condensed or fragmented chromatin; and (iv) necrotic cells had uniformly orange–red fluorescing nuclei with no indication of chromatin fragmentation, and the cells were swollen to large size. The results suggested that treatment with the complex **1** caused death of HepG2 cells through mostly apoptosis.

## Conclusion

Two novel mixed-ligand copper(I) complexes [CuCl(PPh_3_)(L)] containing scorpionate-like ligands (**1**: L = 4-carboxyphenyl)bis(3,5-dimethylpyrazolyl)methane; **2** L = 3-carboxyphenyl)bis(3,5-dimethylpyrazolyl)methane) and PPh_3_ have been synthesised and characterized by using various spectroscopic and analytical methods. The structures of the copper complexes were ascertained by computational method (B3LYP/DFT). *In vitro* DNA binding studies have been performed using various biophysical techniques viz, absorption, fluorescence, and circular dichroism techniques, to envisage their binding site and binding strength; the experimental results revealed significant binding affinity of both complexes **1** and **2** with selective preferentiality of the complex **1** via electrostatic and/or covalent mode. However, the intercalative mode of interaction was also not completely ruled out. We analyzed the electrophoretic pattern of pBR322 DNA upon interaction with **1,** which gives concentration dependent cleavage pattern. The mechanistic study of DNA cleavage with complex **1** is suggestive of hydrolytic as well as oxidative pathways. Also, complex **1** exhibited preferential selectivity towards minor groove of the DNA. These results fortify our idea of intercalative and/or inter-cross linking nature of **1** with DNA. Then molecular modeling studies were carried out to justify our hypothesis of DNA binding affinity of the complex **1**, which corroborated well with above experimental findings. The docking studies of **1** with DNA-topo I and DNA-topo II were analyzed to know the potential of **1** to act as an enzyme inhibitor particularly topo I and topo II. Our results ascertained the potential of **1** to act as topo I inhibitor by blocking the relegation of G11 -OH group and topo II inhibitor by preventing the entry of ATP. Complex **1** was tested against HepG2 human hepatocarcinoma cell line for anticancer activity. *In vitro* cytotoxicity study strongly suggests that complex **1** would bind DNA-Topo complex in a specific mode so as to damage the DNA and/or perturb the replication of DNA and, thus, kill the cancer cells in general and hepatocarcinoma cells in particular. Further studies are required to strongly recommend the preclinical study of complex **1** as a cancer chemotherapeutic.

## Experimental Section

### Materials

3,5-dimethylpyrazole, CuCl, agarose, ascorbic acid, sodium azide (NaN_3_), DMSO, superoxide dismutase (SOD), methyl green, Tris(hydroxymethyl)aminomethane, disodium salt of calf thymus DNA (highly polymerized, stored at 4 °C), 4,6-diamino-phenylindole (DAPI), (Sigma-Aldrich), COCl_2_ (Fluka), H_2_O_2_ (Merck) and pBR322 supercoiled plasmid DNA (Genei) were used as received. The ligands L_A_ and L_**B**_ were prepared by the procedure adopted by Carrano *et al*.^48^,^49^.

### Methods and Instrumentation

Microanalyses (C, H, and N) were carried out with a Carlo Erba Analyzer Model 1108. Molar conductance was measured at room temperature on a Eutech CON 510 conductivity bridge. IR spectra were recorded from 4000 to 100 cm^−1^ with a Perkin-Elmer System 2000 FT-IR instrument. Positive electrospray mass spectra were obtained with a Series 1100 MSI detector HP spectrometer, using MeOH as the mobile phase. Solutions for electrospray ionization mass spectrometry (ESI-MS) were prepared using reagent-grade methanol and the obtained data (masses and intensities) were compared with those calculated by using the IsoPro isotopic abundance simulator, version 2.1. Elemental analyses (C, H, N) were performed with a Fisons Instruments 1108 CHNS-O elemental analyzer. Electronic spectra were recorded on UV-1700 PharmaSpec UV-vis spectrophotometer (Shimadzu). Emission spectra were determined with a Hitachi F-2500 fluorescence spectrophotometer. ^1^H NMR was recorded on Mercury-400BB and ^13^C NMR was recorded on Bruker Avance II 400 NMR spectrometer at 25 °C. Cleavage experiments were performed with the help of Axygen electrophorEsis supported by Genei power supply with a potential range of 50–500 Volts, visualized and photographed by Vilber-INFINITY gel documentation system.

The density functional theory (DFT) calculations were done with the ORCA computational programme^66^,^67^. The geometry optimizations were carried out by hybrid B3LYP functional using the Ahlrichs def2–TZVP basis set for metals and def2–SVP basis set for C, H, O, N atoms^68^,^69^,^70^,^71^,^72^,^73^,^74^,^75^,^76^. The optimized structures were further recalculated using def2–TZVP basis set for all atoms. To accelerate the calculations we utilized the resolution of identity approximation with the decontracted auxiliary def2–TZV/J Coulomb fitting basis sets and the chain–of–spheres approximation to exact exchange as implemented in ORCA^74^,^77^.

The molecular docking studies have been performed by using Hex 8.0.0^78^. All rotatable bonds within the ligand were allowed to rotate freely, and receptor was considered rigid. The crystal structure of the B–DNA dodecamer d(CGCGAATTCGCG)_2_ (PDB ID: 1BNA), human-DNA-Topo-I (PDB ID: 1SC7) and human Topo-IIα (PDB ID: 1ZXM) were retrieved from the protein data bank (http://www.rcsb.org./pdb). Visualization of minimum energy favorable docked poses has been performed using Discovery Studio 4.1 and PyMol^79^,^80^.

DNA binding experiments that included spectral absorption studies, and fluorescence, conformed to the standard methods^50^,^54^,^81^ and practices previously adopted by our laboratory^82^,^83^. Standard error limits were estimated using all data points. Circular dichroic spectra of DNA were obtained by using Circular Dichroism (CD) Spectrometer with Stop Flow-*Applied PhotoPhysicsChirascan*. All experiments were done using 200 μL quartz cell. Each CD spectrum was collected after averaging over at least 3 accumulations using a scan speed of 100 nm min^−1^ and a 5 s response time. Machine plus cuvette baselines were subtracted, and the resultant spectra zeroed outside the absorption bands.

*In vitro* anticancer experiments were carried out by standard protocol with slight modification as reported by us^63^.

### Synthesis of[CuCl(PPh_3_) (L_A_)]

A solution containing CuCl (0.099 g, 1.0 mmol), PPh_3_ (0.262 g, 1.0 mmol) in 5 mL of CH_3_CN was stirred when warmed for 1 h. Then the ligand (L_A_) (0.324 g, 1 mmol) in 10 mL of CH_3_OH was added and allowed stirred for 4 h, at an ambient temperature. The resulting solution was kept for slow evaporation. A colorless crystalline precipitate appeared after 2 days. Yield: (0.195 g, 62%). M.p.192 °C. Anal. Calc. for C_36_H_35_CuN_4_O_2_PCl: C, 63.06; H, 5.10; N, 8.17. Found: C, 62.56; H, 5.08; N, 7.89%.IR (cm^−1^): 2957, 2918, 1716, 1559, 1434, 1425, 1385, 1250, 1109, 1093, 860, 791, 745, 691, 518, 490, 431, 402. ^1^H NMR (CD_3_OD, δ, 293 K): 8.01 (s, 1 H), 7.45 (d, 2 H, ^3^*J*^H-H^ = 8.0 Hz), 7.45, 7.38, and 7.16 (m, 15 H, PC_18_H_15_), 6.62 (d, 1 H, ^3^*J*^H-H^ = 8.1 Hz), 6.24 (s, 2 H, PzCH), 2.61 (s, 6 H, CH_3_Pz), 2.06 (s, 6 H, CH_3_ Pz). ^31^P NMR (CD_3_OD, δ, 293 K): 3.96. ESI-MS {in MeOH, +ve} (obsd (calcd), rel. Intens., assignments): 649.3 (650.2), 100, [C_36_H_35_CuN_4_O_2_P]^+^− 1 H^+^.

### Synthesis of[CuCl(PPh_3_) (L_B_)]

A solution containing CuCl (0.099 g, 1.0 mmol), PPh_3_ (0.262 g, 1.0 mmol) in 5 mL of CH_3_CN was stirred with warming for 1 h. Then the ligand (L_B_) (0.324 g, 1 mmol) in 10 mL of CH_3_OH was added and stirred for 4 h, at the ambient temperature. The resulting solution was kept for slow evaporation. A colorless crystalline precipitate appeared after 4 days. Yield: (0.182 g, 58%). M.p.235 °C. Anal. Calc. for C_36_H_35_CuN_4_O_2_PCl + H_2_O: C, 61.45; H, 5.26; N, 7.96. Found: C, 61.42; H, 4.95; N, 8.36%.IR (cm^−1^): 3047, 2916, 1706, 1558, 1433, 1383, 1221, 1182, 1094, 859, 780, 741, 692, 516, 491, 436, 414. ^1^H NMR (CD_3_OD, δ, 293 K): 8.04 (s, 1 H), 7.61 (d, 1 H, ^3^*J*^H-H^ = 6.8 Hz),7.51, 7.49, 7.29 (m, 15 H, PC_18_H_1′5_), 7.42 (s, 1 H), 6.82 (d, 1 H, ^3^*J*^H-H^ = 7.2 Hz), 6.27 (s, 2 H, PzCH), 2.60 (s, 6 H, CH_3_Pz), 2.13 (s, 6 H, CH_3_ Pz).^31^P NMR (CD_3_OD, δ, 293 K): 4.58. ESI-MS {in MeOH, +ve} (obsd (calcd), rel. Intens., assignments): 649.3 (650.2), 100, [C_36_H_35_CuN_4_O_2_P]^+^- 1 H^+^.

### Anticancer Activity Studies

#### MTT assay

The complex **1** (STR2) was dissolved in minimum quantity of DMSO, diluted in culture medium and used to treat HepG2 hepatocellular carcinoma cells, in 96 well culture plates, at a concentration of the complex ranging from 1 to 10 μM for a period of 24 h. Cisplatin was used as the reference drug, and DMSO was used as the solvent control. A miniaturized viability assay using 3-(4,5-di-methyithiazol-2-yl)-2,5-diphenyl-2H-tetrazolium bromide (MTT) was carried out according to the method described by Mosman (1983). The cells were assayed by the addition of 20 μL of the MTT solution (5 βmg mL-1) prepared in phosphate-buffered saline (PBS). The plates were wrapped in aluminum foil and incubated for 4 h at 37 °C. The purple formazan product was dissolved by the addition of 100 μL of 100% DMSO to each well. The absorbance was monitored at 570 nm (measurement) and 630 nm (reference) using a 96 well plate reader (Bio-Rad, Hercules, CA, USA). Data were collected for triplicates and used to calculate the respective means. The percentage inhibition was calculated from this data using the formula:


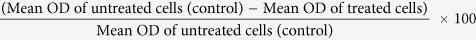


The IC_50_ value was determined as the concentration of the complex/cisplatin that is required to decrease the absorbance to half that of the control.

#### Hoechst 33258 staining

The HepG2 cells were seeded in 6-well plates and allowed to reach 80% confluence. The cells were then treated with the IC_50_ concentration of complex **1** and incubated for 24 h. The cells were trypsinized and pelleted and then suspended in PBS. The suspension of HepG2 was also stained with Hoechst 33258 (Sigma Chemical Co., St. Louis, MO, USA) and incubated at 37 °C for 15 min. At random, 300 cells were observed in the fluorescent microscope at x400 magnification and the percentage of cells reflecting pathological changes was calculated.

#### Acridine orange (AO) and ethidium bromide (EB) staining

A drop of cells in suspension from the previous section was placed on a glass slide and stained with AO and EB (1% in PBS, separately; mixed in 1: 1 ratio) (Sigma Chemical Co., St. Louis, MO, USA), and a cover slip was placed over it to reduce light diffraction. At random, 300 cells were observed in a fluorescent microscope (Carl Zeiss, Jena, Germany) fitted with a 377–355 nm filter and examined at x400 magnification. The percentage of cells reflecting pathological changes was calculated.

## Additional Information

**How to cite this article:** Khan, R. A. *et al*. Heteroleptic Copper(I) Complexes of “Scorpionate” Bis-pyrazolyl Carboxylate Ligand with Auxiliary Phosphine as Potential Anticancer Agents: An Insight into Cytotoxic Mode. *Sci. Rep.* **7**, 45229; doi: 10.1038/srep45229 (2017).

**Publisher's note:** Springer Nature remains neutral with regard to jurisdictional claims in published maps and institutional affiliations.

## Supplementary Material

Supplementary Information

## Figures and Tables

**Table 1:** Some selected experimental and calculated wavenumbers of complexes **1** and **2.**

Vibrational Band	Complex 1	Complex 1	Complex 2	Complex 2
	Experimental (cm^−1^)	Calculated (cm^−1^)	Experimental (cm^−1^)	Calculated (cm^−1^)
v(O-H) stretching	3047	3079	3097	3109
v(Ar-CH) stretching	2916	2934	2957	2994
v(-CH_3_) symmetrical stretching	2829	2824	2918	2965
v(C = O) symmetrical stretching	1706	1707	1715	1729
v(C-O-H) anti-symmetrical stretching	1383	1359	1385	1375
v(C-N) stretching	1251	1263	1250	1264

**Table 2:** Non-covalent interactions of complexes **1** and **2** with the DNA.

Name	Distance (Å)	Category	Type
A:DG12:OP1 - :Complex **1**	4.65	Electrostatic	Pi-Anion
B:DG16 - :Complex **1**	5.23	Hydrophobic	Pi-Pi Stacked
B:DG14 - :Complex **1**	4.82	Hydrophobic	Pi-Pi T-shaped
A:DG10 - :Complex **1**	4.53	Hydrophobic	Pi-Alkyl
A:DC11- : Complex **1**	4.43		Pi-Alkyl
B:DC15 - : Complex **1**	3.65		Pi-Alkyl
B:DG16 - : Complex **1**	3.08		Pi-Alkyl
B:DG16 - : Complex **1**	4.67		Pi-Alkyl
B:DG16 - : Complex **1**	3.58		Pi-Alkyl
B:DC23:OP1 - :Complex **2**	3.52	Electrostatic	Pi-Anion
B:DC21 - :Complex **2**	5.28	Hydrophobic	Pi-Alkyl
B:DG22 - : Complex **2**	4.64		Pi-Alkyl
B:DG22 - : Complex **2**	4.38		Pi-Alkyl
B:DG16 - : Complex **2**	3.58		Pi-Alkyl
B:DC23 - : Complex **2**	4.63		Pi-Alkyl

**Table 3:** Non-covalent interactions of complexes **1** and **2** with the Human–DNA–Topo–I.

Name	Distance (Å)	Category	Type
D:Complex **1**:H - B:DT10:O2	2.89	Hydrogen Bond	Conventional
B:DT10 - D: Complex **1**	4.26		Pi-Pi T-shaped
C:DG12 - D: Complex **1**	4.96		Pi-Pi T-shaped
D:DA113 - D: Complex **1**	5.20		Pi-Pi T-shaped
D:Complex **1**:C - A:LEU721	5.43		Alkyl
B:DT10 - D: Complex **1**:C	5.48	Hydrophobic	Pi-Alkyl
C:DG12 - D: Complex **1**:C	2.70		Pi-Alkyl
C:DG12 - D: Complex **1**:C	5.20		Pi-Alkyl
C:DG12 - D: Complex **1**:C	3.16		Pi-Alkyl
D:DA113 - D:Complex **2**	5.58		Pi-Pi Stacked
C:DG12 - D: Complex **2**	4.26		Pi-Pi T-shaped
D: Complex **2** - C:DG12	3.93		Pi-Pi T-shaped
C:DG12 - D: Complex **2**:C	2.47		Pi-Alkyl
C:DG12 - D: Complex **2**:C	3.55	Hydrophobic	Pi-Alkyl
C:DG12 - D: Complex **2**:C	4.72		Pi-Alkyl
D:DC111 - D: Complex **2**:C	5.07		Pi-Alkyl
D:DC112 - D: Complex **2**:C	3.68		Pi-Alkyl
D:DA113 - D: Complex **2**:C	4.22126		Pi-Alkyl

**Table 4:** Non-covalent interactions of complex **2** with the Topo–II.

Name	Distance (Å)	Category	Type
A:ARG98:NH2 - : Complex **1**	3.75	Electrostatic	Pi-Cation
A:HIS130 - : Complex **1**	5.20		Pi-Pi T-shaped
Complex 1:C - A:PRO126	3.95	Hydrophobic	Alkyl
Complex **1**:C - A:VAL137	3.05		Alkyl
Complex **1** - A:ARG98	5.37		Pi-Alkyl
Complex **1** - A:PRO126	5.30		Pi-Alkyl
Complex **1** - A:LEU140	5.36		Pi-Alkyl
Complex **2**:C - A:MET135	3.63		Alkyl
A:TRP194 - : Complex **2**:C	4.95	Hydrophobic	Pi-Orbitals
Complex **2**:C - A:MET135	5.43		Pi-Orbitals
Complex **2** - A:ALA201	4.49		Pi-Orbitals

**Table 5:** *In vitro* cytotoxicity assay for complexes **1**, **2** and cisplatin against HepG2 human hepatocellular carcinoma cells.

Synthetic complex/Drug standard	IC_50_ for 24 h (μM)
Complex **1**	3.3 ± 0.02
Complex **2**	na*
Cisplatin	7.2 ± 0.04

^*^na means not active.

## References

[b1] World Cancer Report 2014, World Health Organization, pp. Chapter 1.1. and 5.6. ISBN 9283204298 (2014).

[b2] Siegel R. L., Miller K. D. & Jemal A. Cancer Statistics, 2016. CA Cancer J. Clinicians, 66, 7–30 (2016). 10.3322/caac.21332 26742998

[b3] Rosenberg B., VanCamp L., Trosko J. E. & Mansour V. H. Platinum compounds: a new class of potent antitumour agents. Nature, 222, 385–386 (1969). 5782119 10.1038/222385a0

[b4] Rosenberg B. Platinum complexes for the treatment of cancer. Interdiscip. Sci. Rev. 3, 134–147 (1978).

[b5] Galanski M., Arion V. B., Jakupec M. A. & Keppler B. K. Recent developments in the field of tumor-inhibiting metal complexes Curr. Pharm. Des. 9, 2078–2089 (2003). 14529417 10.2174/1381612033454180

[b6] Rosenberg B. Noble metal complexes in cancer chemotherapy. Adv. Exp. Med. Biol. 91, 129–150 (1977). 343531 10.1007/978-1-4684-0796-9_10

[b7] Hill J. M. & Speer R. J. Organo-Platinum Complexes as Antitumor Agents. Anticancer Res. 2, 173–186 (1982). 6751211

[b8] Khan R. A. . Carbohydrate linked Organotin (IV) Complexes as Human Topoisomerase Iα inhibitor and their antiproliferative effects against human carcinoma cell line (Huh7) by transcriptional regulation of specific gene. Dalton Trans. 43, 2534–2548 (2014). 24310209 10.1039/c3dt51973b

[b9] Mjos K. D. & Orvig C. Metallodrugs in Medicinal Inorganic Chemistry, Chem. Rev. 114, 4540–4563 (2014). 24456146 10.1021/cr400460s

[b10] Gasser G., Ott I. & Metzler-Nolte N. Organometallic Anticancer Compounds, J. Med. Chem. 54(1), 3–25 (2011). 21077686 10.1021/jm100020w PMC3018145

[b11] Frezza M. . Novel Metals and Metal Complexes as Platforms for Cancer Therapy, Curr Phar. Des. 16(16), 1813–1825 (2010). 10.2174/138161210791209009 PMC3759287 20337575

[b12] Allardyce C. S. & Dyson P. J. Metal-based drugs that break the rules, Dalton Trans. 45, 3201–3209 (2016). 26820398 10.1039/c5dt03919c

[b13] Santini C. . Advances in Copper Complexes as Anticancer Agents, Chem. Rev. 114(1), 815–862 (2014). 24102434 10.1021/cr400135x

[b14] Suss-Fink G. Arene ruthenium complexes as anticancer agents. Dalton Trans. 1673–1688 (2010). 20449402 10.1039/b916860p

[b15] Che C. M. & Sun R. W. Y. Therapeutic applications of gold complexes: lipophilic gold(III) cations and gold(I) complexes for anti-cancer treatment Chem. Commun. 47, 9554–9560 (2011). 10.1039/c1cc10860c 21674082

[b16] Barry N. P. E. & Sadler P. J. Exploration of the medical periodic table: towards new targets Chem. Commun. 49, 5106–5131 (2013). 10.1039/c3cc41143e 23636600

[b17] Trondl R. . NKP-1339, the first ruthenium-based anticancer drug on the edge to clinical application Chem. Sci. 5, 2925–2932 (2014).

[b18] Kapdi A. R. & Fairlamb J. S. Anti-cancer palladium complexes: a focus on PdX2L2, palladacycles and related complexes Chem. Soc. Rev. 43, 4751–4777 (2014). 24723061 10.1039/c4cs00063c

[b19] Hadjikakou S. K. & Hadjiliadis N. Antiproliferative and anti-tumor activity of organotin compounds, Coord. Chem. Rev. 253, 235–249 (2009).

[b20] Baul T. S. B. . Molecular basis of the interaction of novel tributyltin(IV) 2/4-[(E)-2-(aryl)-1-diazenyl]benzoates endowed with an improved cytotoxic profile: Synthesis, structure, biological efficacy and QSAR studies J. Inorg. Biochem. 104, 950 (2010). 20553814 10.1016/j.jinorgbio.2010.05.001

[b21] Baul T. S. B. . An *in vitro* comparative assessment with a series of new triphenyltin(IV) 2-/4-[(E)-2-(aryl)-1-diazenyl]benzoates endowed with anticancer activities: Structural modifications, analysis of efficacy and cytotoxicity involving human tumor cell lines J. Inorg. Biochem. 107, 119–128 (2012). 22182574 10.1016/j.jinorgbio.2011.10.008

[b22] Wojciech P. & Nicholas M. D. J. D. Molecular characterization of a copper-containing nitrite reductase from Rhodopseudomonas sphaeroides forma sp. Denitrificans BBA-Protein Struct. Mol. Enzymol. 828, 130 (1985).

[b23] Chang C. M. . Kinetic and structural analysis of substrate specificity in two copper amine oxidases from Hansenula polymorpha Biochemistry 49, 2540–2550 (2010). 20155950 10.1021/bi901933d PMC2851405

[b24] Tainer J. A., Getzoff E. D., Richardson J. S. & Richardson D. C. Structure and mechanism of copper, zinc superoxide dismutase Nature 306, 284–287 (1983). 6316150 10.1038/306284a0

[b25] Solomon E. I., Chen P., Metz M., Lee S.-K. & Palmer A. E. Oxygen Binding, Activation, and Reduction to Water by Copper Proteins Angew. Chem., Int. Ed. 40, 4570–4590 (2001). 10.1002/1521-3773(20011217)40:24<4570::aid-anie4570>3.0.co;2-4 12404359

[b26] Matoba Y., Kumagai T., Yamamoto A., Yoshitsu H. & Sugiyama M. Crystallographic Evidence That the Dinuclear Copper Center of Tyrosinase Is Flexible during Catalysis, J. Biol. Chem. 281, 8981–8990 (2006). 16436386 10.1074/jbc.M509785200

[b27] Marzano C., Pellei M., Tisato F. & Santini C. Copper complexes as anticancer agents Anti-Cancer Agents in Medicinal Chemistry 9, 185–211 (2009). 19199864 10.2174/187152009787313837

[b28] Sabounchei S. J. . Synthesis and structural characterization of dimeric phosphine ylide Cu(I) complexes: Application in Suzuki cross-coupling reactions and biological evaluation as antibacterial agents. J. Organomet. Chem. 761, 111–119 (2014).

[b29] Starosta R., Florek M., Król J. Puchalska M. & Kochel A. Copper(I) iodide complexes containing new aliphatic aminophosphine ligands and diimines luminescent properties and antibacterial activity New J. Chem. 34, 1441–1449 (2010).

[b30] Fujimori Y. ., Improvement of the redox balance increases L-valine production by Corynebacterium glutamicum under oxygen deprivation conditions Appl. Environ. Microbiol. 78, 4951–955 (2012). 10.1128/AEM.07056-11 PMC3264131 22138982

[b31] Komarnicka U. K., Starosta R., Kyzioł A. & Jeżowska-Bojczuk M. Copper(I) complexes with phosphine derived from sparfloxacin. Part I – structures, spectroscopic properties and cytotoxicity, Dalton Trans. 44, 12688–12699 (2015). 26085118 10.1039/c5dt01146a

[b32] Gandin V. . Homoleptic phosphino copper(I) complexes with *in vitro* and *in vivo* dual cytotoxic and anti-angiogenic activity Metallomics 7, 1497–1507 (2015). 26190698 10.1039/c5mt00163c

[b33] Plotek M., Dudek K. & Kyziol A. Selected copper(I) complexes as potential anticancer agent. CHEMIK 6712, 1181–1190 (2013).

[b34] Varna D., Hatzidimitriou A. G., Velali E., Pantazaki A. A. & Aslanidis A. Structural diversity in dinuclear copper(I) halide complexes of 2,4-dithiouracil: Synthesis, crystal structures, induction of DNA damage and oxidative stress mediated by ROS,P. Polyhedron 88, 40–47 (2015).

[b35] Komarnicka U. K. . Copper(I) complexes with phosphine derived from sparfloxacin. Part II: a first insight into the cytotoxic action mode Dalton Trans. 45, 5052–5063 (2016). 26674970 10.1039/c5dt04011f

[b36] Krajewska B. Mono- (Ag, Hg) and di- (Cu, Hg) valent metal ions effects on the activity of jack bean urease. Probing the modes of metal binding to the enzyme J. Enzyme Inhib. Med. Chem. 23(4), 535–42 (2008). 18608777 10.1080/14756360701743051

[b37] Santini C. . New copper(I) phosphane complexes of dihydridobis(3-nitro-1,2,4-triazolyl)borate ligand showing cytotoxic activity Inorg. Chim. Acta 357(12), 3549–3555 (2004). 10.1016/j.jinorgbio.2005.11.014 16387362

[b38] Gandin V. . *In Vitro* and *in Vivo* Anticancer Activity of Copper(I) Complexes with Homoscorpionate Tridentate Tris(pyrazolyl)borate and Auxiliary Monodentate Phosphine Ligands J. Med. Chem. 57(11), 4745–4760 (2014). 24793739 10.1021/jm500279x

[b39] Iakovidis I., Delimaris I. & Piperakis S. M. Copper and Its Complexes in Medicine: A Biochemical Approach. Mol. Biol. Internat. 2011, Article ID 594529, 13 pages (2011). 10.4061/2011/594529 PMC3195324 22091409

[b40] Gordon D. A. In: Kelley W. N., Harris E. D., Ruddy S., Sledge S. B., editors. Textbook of Rheumatology(IV ed.), Philadelphia, W.B. Saunders Company, p. 805–23 (1989).

[b41] Shaw C. F. Gold-based therapeutic agents. Chem. Rev. 99, 2589–2600 (1999). 11749494 10.1021/cr980431o

[b42] Fan C. . Enhancement of auranofin-induced lung cancer cell apoptosis by selenocystine, a natural inhibitor of TrxR1 *in vitro* and *in vivo*, Cell Death and Disease 5, e1191 (2014). 24763048 10.1038/cddis.2014.132 PMC4001298

[b43] Roder C. & Thomson M. J. Auranofin: repurposing an old drug for a golden new age, R D., Drugs 15(1), 13–20 (2015). 10.1007/s40268-015-0083-y PMC4359176 25698589

[b44] Tisato F. . Insights into the cytotoxic activity of the phosphane copper(I) complex [Cu(thp)_4_][PF_6_]. J. Inorg. Biochem. http://dx.doi.org/10.1016/j.jinorgbio.2016.07.007 (2016). 10.1016/j.jinorgbio.2016.07.007 27449160

[b45] Special Issue: *Eur. J. Inorg. Chem.,* edited by Pettinari, C. The Significance of Scorpionate Ligands 50 Years On (Cluster Issue), **15-16**, 2209-2657 (2016).

[b46] Pettinari C. & Scorpionates I. I.: Chelating Borate Ligands Dedicated to Swiatoslaw Trofimenko, Imperial College Press, London (2008).

[b47] Khan R. A. . Organometallic ruthenium(II) scorpionate as topo IIα inhibitor; *in vitro* binding studies with DNA, HPLC analysis and its anticancer activity. J. Organomet. Chem. 771, 47–58 (2014).

[b48] Santillan G. A. & Carrano C. J. Synthesis and Characterization of Copper(II) Complexes of Nonfacially Coordinating Heteroscorpionate Ligands (4-Carboxyphenyl)bis(3,5-dimethylpyrazolyl)methane and (3-Carboxyphenyl)bis(3,5-dimethylpyrazolyl)methane. Inorg. Chem. 46, 1751–1759 (2007). 17286399 10.1021/ic062226u

[b49] Santillan G. A. & Carrano C. J. Cobalt, Zinc, and Nickel Complexes of a Diatopic Heteroscorpionate Ligand: Building Blocks for Coordination Polymers. Inorg. Chem. 47(3), 930–939 (2008). 18186625 10.1021/ic701718b

[b50] Lakowicz J. R. & Weber G. Quenching of fluorescence by oxygen. Probe for structural fluctuations in macromolecules. Biochemistry 12, 4161–4170 (1973). 4795686 10.1021/bi00745a020 PMC6959846

[b51] Folda A. . Insights into the strong *in*-*vitro* anticancer effects for bis(triphenylphosphane)iminium compounds having perchlorate, tetrafluoridoborate and bis(chlorido)argentate anions. J. Inorg. Biochem. 153 346–354 (2015). 26384162 10.1016/j.jinorgbio.2015.08.030

[b52] Garbett N. C., Ragazzon P. A. & Chaire J. B. Circular dichroism to determine binding mode and affinity of ligand–DNA interactions. Nature Protocols 2, 3166–3172 (2007). 18079716 10.1038/nprot.2007.475

[b53] Villarreal W. . Chiral Platinum(II) Complexes Featuring Phosphine and Chloroquine Ligands as Cytotoxic and Monofunctional DNA-Binding Agents. Inorg. Chem. 54(24), 11709–11720 (2015). 26606142 10.1021/acs.inorgchem.5b01647

[b54] Wolfe A., Shimmer G. H. & Meehan T. Polycyclic aromatic hydrocarbons physically intercalate into duplex regions of denatured DNA. Biochemistry 26, 6392 (1987). 3427013 10.1021/bi00394a013

[b55] Yousuf I. . Mechanistic insights of a novel chromone–appended Cu(II) anticancer drug entity: *In vitro* binding profile with DNA/RNA substrates and cytotoxic activity against MCF-7 and HepG2 cancer cells. Dalton Trans. 44(22), 10330–10342 (2015). 25970097 10.1039/c5dt00770d

[b56] Tabassum S. . Chiral Heterobimetallic Complexes Targeting Human DNA-Topoisomerase Iα. Dalton Trans. 42, 16749 (2013). 24077532 10.1039/c3dt51209f

[b57] Scolaro C. . *In vitro* and *in vivo* evaluation of ruthenium(II)-arene PTA complexes. J. Med. Chem. 48, 4161–4171 (2005). 15943488 10.1021/jm050015d

[b58] Gossens C., Tavernelli I. & Rothlisberger U. DNA Structural Distortions Induced by Ruthenium−Arene Anticancer Compounds. J. Am. Chem. Soc. 130, 10921–10928 (2008). 18651736 10.1021/ja800194a

[b59] Tabassum S., Khan R. A., Arjmand F., Juvekar A. S. & Zingde S. M. Synthesis of carbohydrate-conjugate heterobimetallic Cu^II^-Sn_2_^IV^ and Zn^II^-Sn_2_^IV^ complexes; their interactions with CT DNA and nucleotides; DNA cleavage, *in-vitro* cytotoxicity. Eur. J. Med. Chem. 45, 4797–4806 (2010). 20813437 10.1016/j.ejmech.2010.07.046

[b60] Jaividhya P., Ganeshpandian M., Dhivya R., Akbarsha M. A. & Palaniandavar M. Fluorescent mixed ligand copper(II) complexes of anthracene-appended Schiff bases: studies on DNA binding, nuclease activity and cytotoxicity. Dalton Trans. 44, 11997–12010 (2015). 26076117 10.1039/c5dt00899a

[b61] Tsiaggali M. A., Andreadou E. G., Hatzidimitriou A. G., Pantazaki A. A. & Aslanidis P. Copper(I) halide complexes of N-methylbenzothiazole-2-thione: Synthesis, structure, luminescence, antibacterial activity and interaction with DNA. J. Inorg. Biochem. 121, 121–128 (2013). 23376553 10.1016/j.jinorgbio.2013.01.001

[b62] Khan R. A. . Crystal Structure of heterobimetallic Ruthenium (II)-Tin(IV) complex; *in vitro* DNA Binding Studies with CT-DNA, Topo I Inhibition activity and DFT Calculations. Organometallics. 32(9), 2546–2551 (2013).

[b63] Tabassum S. . Chiral Heterobimetallic Complexes Targeting Human DNA-Topoisomerase Iα. RSC Adv. 5, 47439–47450 (2015). 10.1039/c3dt51209f 24077532

[b64] Burrows C. J. & Muller J. G. Oxidative Nucleobase Modifications Leading to Strand Scission. Chem. Rev. 98, 1109 (1998). 11848927 10.1021/cr960421s

[b65] Selvakumar B., Rajendiran V., Maheswari P. U., Evans H. S. & Palaniandavar M. Structures, spectra, and DNA-binding properties of mixed ligand copper (II) complexes of iminodiacetic acid: The novel role of diimine co-ligands on DNA conformation and hydrolytic and oxidative double strand DNA cleavage. J. Inorg. Biochem. 100, 316–330 (2006). 16406550 10.1016/j.jinorgbio.2005.11.018

[b66] Neese F. The ORCA program system. WIREs Comput. Mol. Sci. 2, 73–78 (2012).

[b67] Neese F. O. R. C. A. An ab Initio. Density Functional and Semi empirical Program Package version” (2009).

[b68] Lee C., Yang W. & Parr R. G. Development of the Colle-Salvetti correlation-energy formula into a functional of the electron density. Phys. Rev. B. 37, 785–789 (1988). 10.1103/physrevb.37.785 9944570

[b69] Weigend F. & Ahlrichs R. Balanced basis sets of split valence, triple zeta valence and quadruple zeta valence quality for H to Rn: Design and assessment of accuracy. Phys. Chem. Chem. Phys. 7, 3297–3305. (2005). 16240044 10.1039/b508541a

[b70] Schaefer A., Huber C. & Ahlrichs R. Fully optimized contracted Gaussian basis sets of triple zeta valence quality for atoms Li to Kr. J. Chem. Phys. 100, 5829–5835 (1994).

[b71] Schaefer A., Horn H. & Ahlrichs R. Fully optimized contracted Gaussian basis sets for atoms Li to Kr. J. Chem. Phys. 97, 2571–2577 (1992).

[b72] Grimme S., Antony J., Ehrlich S. & Krieg H. A consistent and accurate *ab initio* parametrization of density functional dispersion correction (DFT-D) for the 94 elements H-Pu. J. Chem. Phys. 132, 154104 (2010). 20423165 10.1063/1.3382344

[b73] Steffen C., Thomas K., Huniar U., Hellweg A., Rubner O. & Schroer A. TmoleX—A graphical user interface for TURBOMOLE, J. Comput. Chem. 31, 2967–2970 (2010). 20928852 10.1002/jcc.21576

[b74] Schaefer A., Horn H. & Ahlrichs R. Fully optimized contracted Gaussian basis sets for atoms Li to Kr. J. Chem. Phys. 97, 2571−2577 (1992).

[b75] Ahlrichs R. . The Ahlrichs auxiliary basis sets were obtained from the Turbo Mole basis set library under ftp.chemie.uni-karlsruhe.de/pub/jbasen, unpublished results.

[b76] Ruiz E., Cano J., Alvarez S. & Alemany P. Broken symmetry approach to calculation of exchange coupling constants for homobinuclear and heterobinuclear transition metal complexes. J. Comp. Chem. 20, 1391−1400 (1999).

[b77] Onofrio N. & Mouesca J. M. Inorg. Chem. 50, 5577−5886 (2011). 21618973 10.1021/ic200198f

[b78] Mustard D. & Ritchie D. W. Docking essential dynamics eigenstructures. Proteins Struct. Funct. Bioinf. 60, 269 (2005). 10.1002/prot.20569 15981272

[b79] Accelrys Software Inc., Discovery Studio Modeling Environment, Release 4.0, San Diego: Accelrys Software Inc. (2013).

[b80] The PyMOL Molecular Graphics System, Version 1.5.0.4 Schrödinger, LLC.

[b81] Reichmann M. E., Rice S. A., Thomas C. A. & Doty P. A Further Examination of the Molecular Weight and Size of Desoxypentose Nucleic Acid J. Am. Chem. Soc. 76, 3047–3053 (1954).

[b82] Tabassum S., Al-Asbahy W. M., Afzal M., Arjmand F. & Khan R. H. Interaction and photo-induced cleavage studies of a copper based chemotherapeutic drug with human serum albumin: spectroscopic and molecular docking study. Molecular BioSystems. 8(9), 2424–2433 (2012). 22790833 10.1039/c2mb25119a

[b83] Tabassum S., Zaki M., Afzal M. & Arjmand F. New modulated design and synthesis of quercetin–Cu^II^/Zn^II^–Sn_2_^IV^ scaffold as anticancer agents: *in vitro* DNA binding profile, DNA cleavage pathway and Topo-I activity. Dalton Trans. 42(27), 10029–10041 (2013). 23715526 10.1039/c3dt50646k

