# Supplementary material for: Heteroleptic Copper(I) Complexes of “Scorpionate” Bis-pyrazolyl Carboxylate Ligand with Auxiliary Phosphine as Potential Anticancer Agents: An Insight into Cytotoxic Mode
Source: Sci Rep. 2017 Mar 24;7:45229. doi: 10.1038/srep45229 (PMC5364558; doi:10.1038/srep45229)
Supplement: Supplementary Information [file srep45229-s1.doc]

***Supporting Information to the Paper:***

**Heteroleptic Copper(I) Complexes of “Scorpionate” Bis-pyrazolyl Carboxylate Ligand with Auxiliary Phosphine as Potential Anticancer Agents: An Insight into Cytotoxic Mode**

Rais Ahmad Khan,^Φ^, Mohammad Usman,^‡^ Dhivya Rajakumar,^†^ Perumalsamy Balaji,^†^ Ali Alsalme,^Φ^ Hamad Al-Lohedan,^Φ,₸^ Farukh Arjmand,^‡^ Khalid Al Farhan,^Φ^ Mohammad Abdulkader Akbarsha,^†^ Fabio Marchetti,^$^ Claudio Pettinari,^$^ and Sartaj Tabassum^₸,Φ,‡^,*.

*^₸^Surfactant Research Chair, ^Φ^Department of Chemistry, College of Science, King Saud University, Riyadh 11451, Saudi Arabia.*

*^‡^Department of Chemistry, Aligarh Muslim University, Aligarh-202002, India*

*^†^National Center for Alternatives for Animal Experiments, Bharathidasan University, Tiruchirappalli, 620024, India.*

*^$^Dipartimento di Scienze Chimiche, Universita´ degli Studi di Camerino, via S. Agostino 1,62032 Camerino, Macerata, Italy.*

** Corresponding Author: Email:* [*tsartaj62@yahoo.com*](mailto:tsartaj62@yahoo.com) *Tel. No. : +91 9358255791.*

**Table of Contents:**

**Table. S1** Selected bond lengths for complexes **1** and **2**.

**Table. S2** Selected bond angles for complexes **1** and **2**.

**Table. S3** B3LYP/DFT optimized structure coordinates of complexes **1.**

**Table. S4** B3LYP/DFT optimized structure coordinates of complex **2**

**Table S5**. Energy of FMOs for complexes **1** and **2**, calculated in the gas phase.

**Figures S1-S4:** IR spectra of Ligands **L1**, **L2**, complex **1** and complex **2**.

**Figures S5-S6:** ^1^H NMR spectra complex **1** and complex **2**.

**Figures S7-S8:** ^31^P NMR spectra of complexes **1** and **2**.

**Figure S9.**Simulated UV-spectra of 1 and 2

**Tables**

**Table. S1** Selected bond lengths for complexes **1** and **2**.

| **Complex 1** | **Bond length (A˚)** | **Complex 2** | **Bond length (A˚)** |
| --- | --- | --- | --- |
| Cu – N1 | 2.148 | Cu – N1 | 2.153 |
| Cu – P1 | 2.176 | Cu – P1 | 2.253 |
| Cu – Cl1 | 2.295 | Cu – Cl1 | 2.279 |
| Cu – N3 | 2.309 | Cu – N3 | 2.107 |

**Table. S2** Selected bond angles for complexes **1** and **2**.

| **Complex 1** | **Bond angle** | **Complex 2** | **Bond angle** |
| --- | --- | --- | --- |
| N1 – Cu – N3 | 90.64 | N1 – Cu – N3 | 93.87 |
| N1 – Cu – Cl1 | 108.73 | N1 – Cu – Cl1 | 111.99 |
| Cl1 – Cu – N3 | 127.53 | Cl1 – Cu – N3 | 122.26 |
| P1 – Cu – N3 | 102.13 | P1 – Cu – N3 | 104.02 |
| N1 – Cu – P1 | 100.25 | N1 – Cu – P1 | 100.49 |
| P1 – Cu – Cl1 | 120.45 | P1 – Cu – Cl1 | 119.37 |

**Table. S3** B3LYP/DFT optimized structure coordinates of complexes **1**

C 0.338844000 1.431336000 -0.758462000

C 0.212075000 0.360426000 -1.628147000

C 1.097150000 -0.642989000 -1.151580000

N 1.733227000 -0.198555000 -0.072309000

N 1.272775000 1.049968000 0.170345000

N 3.933188000 1.141178000 1.606354000

C 5.047445000 1.779476000 1.961533000

C 4.771685000 3.160291000 2.148926000

C 3.419656000 3.319578000 1.895749000

N 2.953709000 2.079689000 1.550375000

C -0.314860000 2.772226000 -0.767545000

C 1.322061000 -2.034558000 -1.644494000

C 2.548648000 4.528261000 1.992921000

C 6.342784000 1.062800000 2.146262000

C 1.896728000 -0.240986000 4.745877000

C 1.953430000 0.789789000 3.811050000

C 1.249695000 0.686350000 2.603768000

C 0.388752000 -0.397572000 2.401799000

C 0.381700000 -1.454187000 3.305042000

C 1.180189000 -1.405135000 4.452955000

C 1.564420000 1.651281000 1.461171000

Cu 3.592882000 -0.845296000 0.786374000

Cl 3.229286000 -2.804493000 1.952169000

P 4.930221000 -0.832855000 -1.079128000

C 4.097289000 0.256832000 -2.305681000

C 5.206422000 -2.436393000 -1.930525000

C 6.657414000 -0.198749000 -1.010084000

C 3.612081000 -0.194771000 -3.541042000

C 2.807531000 0.631839000 -4.333436000

C 2.488783000 1.922478000 -3.908452000

C 2.984752000 2.388669000 -2.685920000

C 3.772600000 1.561971000 -1.887499000

C 6.021196000 -2.539138000 -3.071780000

C 6.272627000 -3.785446000 -3.645508000

C 5.716903000 -4.939695000 -3.078854000

C 4.909581000 -4.842739000 -1.942994000

C 4.653271000 -3.594442000 -1.362759000

C 7.092400000 0.982471000 -1.626267000

C 8.409682000 1.424279000 -1.453898000

C 9.302265000 0.689276000 -0.670331000

C 8.879474000 -0.503828000 -0.072018000

C 7.567391000 -0.943444000 -0.238761000

C 1.373403000 -2.626837000 5.305059000

O 1.306004000 -2.623814000 6.508841000

O 1.643731000 -3.734959000 4.600165000

H -0.428660000 0.311158000 -2.504918000

H 5.470071000 3.937753000 2.449366000

H 0.423474000 3.576697000 -0.927165000

H -1.045760000 2.825702000 -1.585678000

H -0.853875000 2.988654000 0.170346000

H 1.367110000 -2.725548000 -0.789529000

H 0.520110000 -2.341077000 -2.330139000

H 2.275630000 -2.124127000 -2.183027000

H 2.079691000 4.783008000 1.027581000

H 1.741227000 4.393953000 2.732279000

H 3.151208000 5.389093000 2.312779000

H 7.059391000 1.314802000 1.350138000

H 6.801182000 1.345752000 3.107306000

H 6.179686000 -0.023234000 2.133998000

H 2.481192000 -0.191709000 5.666527000

H 2.617209000 1.637712000 3.993735000

H -0.191175000 -0.483190000 1.483482000

H -0.191300000 -2.354394000 3.074872000

H 0.960484000 2.564333000 1.519307000

H 3.845657000 -1.204150000 -3.882653000

H 2.419103000 0.256222000 -5.283469000

H 1.852950000 2.563464000 -4.524859000

H 2.735259000 3.394762000 -2.338429000

H 4.116875000 1.922280000 -0.917078000

H 6.473768000 -1.641039000 -3.499269000

H 6.908257000 -3.861329000 -4.531531000

H 5.928986000 -5.915747000 -3.523621000

H 4.482982000 -5.741040000 -1.489924000

H 4.056896000 -3.521465000 -0.446889000

H 6.404571000 1.565530000 -2.241022000

H 8.738240000 2.347824000 -1.937801000

H 10.325381000 1.044707000 -0.523344000

H 9.574254000 -1.088223000 0.536220000

H 7.235130000 -1.866679000 0.243827000

H 1.992007000 -3.493835000 3.706223000

**Table. S4** B3LYP/DFT optimized structure coordinates of complex **2**

C -7.406910000 0.887660000 -0.589468000

C -7.492472000 -0.179873000 -1.467278000

C -6.528889000 -1.126763000 -1.033648000

N -5.894640000 -0.659234000 0.040559000

N -6.430654000 0.553570000 0.313962000

N -3.712873000 0.789625000 1.722192000

C -2.611854000 1.470812000 2.037675000

C -2.909543000 2.858585000 2.090857000

C -4.257721000 2.974026000 1.794729000

N -4.702188000 1.700506000 1.560888000

C -8.144482000 2.184446000 -0.566057000

C -6.185119000 -2.463347000 -1.605033000

C -5.127622000 4.184263000 1.717155000

C -1.311108000 0.782037000 2.283780000

C -6.614299000 0.610528000 5.248613000

C -6.455001000 1.234331000 4.009726000

C -6.383437000 0.477275000 2.832113000

C -6.477379000 -0.915689000 2.911178000

C -6.624545000 -1.547075000 4.152183000

C -6.705645000 -0.779402000 5.320542000

C -6.091752000 1.243794000 1.541710000

Cu -3.883304000 -1.046829000 0.703378000

Cl -3.708483000 -3.052023000 1.772675000

P -2.748039000 -0.714542000 -1.214609000

C -3.537954000 0.700664000 -2.066819000

C -2.688525000 -2.116635000 -2.394265000

C -0.964020000 -0.294473000 -1.056075000

C -4.473036000 0.536741000 -3.100501000

C -5.219664000 1.623768000 -3.566074000

C -5.047611000 2.892004000 -3.007378000

C -4.123839000 3.064918000 -1.968302000

C -3.387915000 1.979776000 -1.495493000

C -2.381258000 -1.968621000 -3.756111000

C -2.311888000 -3.091613000 -4.582235000

C -2.521076000 -4.370058000 -4.050290000

C -2.800965000 -4.522784000 -2.690184000

C -2.891447000 -3.400687000 -1.861780000

C -0.335984000 0.813664000 -1.641799000

C 1.014073000 1.074938000 -1.383660000

C 1.750069000 0.223806000 -0.554313000

C 1.136662000 -0.904192000 0.003932000

C -0.212408000 -1.158708000 -0.239057000

C -6.775612000 -3.045118000 4.268322000

O -7.374799000 -3.536149000 5.197225000

O -6.265458000 -3.792873000 3.292374000

H -8.158876000 -0.261533000 -2.322859000

H -2.227225000 3.671429000 2.328570000

H -7.458956000 3.033725000 -0.724240000

H -8.888052000 2.202972000 -1.374192000

H -8.683389000 2.347744000 0.382723000

H -5.693874000 -3.080041000 -0.839382000

H -7.086854000 -2.978970000 -1.967321000

H -5.489170000 -2.372765000 -2.454608000

H -5.556883000 4.316177000 0.710522000

H -5.966449000 4.147736000 2.432379000

H -4.531666000 5.076457000 1.952585000

H -0.575611000 1.020043000 1.500466000

H -0.884649000 1.100806000 3.248375000

H -1.461228000 -0.306107000 2.298056000

H -6.671079000 1.213467000 6.157917000

H -6.370084000 2.323705000 3.961213000

H -6.419924000 -1.515984000 2.008228000

H -6.848355000 -1.294432000 6.272031000

H -6.691755000 2.157738000 1.536562000

H -4.632587000 -0.448246000 -3.541138000

H -5.944864000 1.470604000 -4.369175000

H -5.628938000 3.739533000 -3.379072000

H -3.973743000 4.048640000 -1.514722000

H -2.702658000 2.128196000 -0.659770000

H -2.188023000 -0.975905000 -4.170246000

H -2.073671000 -2.971140000 -5.642327000

H -2.455775000 -5.247896000 -4.697959000

H -2.959956000 -5.517385000 -2.265408000

H -3.128282000 -3.515969000 -0.798768000

H -0.902060000 1.486394000 -2.288289000

H 1.492781000 1.947869000 -1.835081000

H 2.798308000 0.443003000 -0.335355000

H 1.707170000 -1.578264000 0.647988000

H -0.701974000 -2.016059000 0.232109000

H -5.519433000 -3.374694000 2.785948000

**Table S5**. Energy of FMOs for complexes **1** and **2**, calculated in the gas phase.

|  | | Complex **1** | Complex **2** |
| --- | --- | --- | --- |
| **Molecular Orbitals** | **MO. No** | **Energy (eV)** | **Energy (eV)** |
| LUMO+4 | 183 | -0.61 | -0.77 |
| LUMO+3 | 182 | -0.70 | -0.82 |
| LUMO+2 | 181 | -0.89 | -0.93 |
| LUMO+1 | 180 | -1.07 | -1.07 |
| LUMO | 179 | -1.32 | -1.55 |
| HOMO | 178 | -5.19 | -5.16 |
| HOMO-1 | 177 | -5.51 | -5.63 |
| HOMO-2 | 176 | -5.85 | -5.91 |
| HOMO-3 | 175 | -6.35 | -6.32 |
| HOMO-4 | 174 | -6.64 | -6.45 |

**Figures**

**Figure S1.** IR spectrum of ligand **L1.**

**Figure S2.** IR spectrum of ligand **L2.**

**Figure S4.** IR spectrum of complex **1.**

**Figure S3.** IR spectrum of complex **2.**


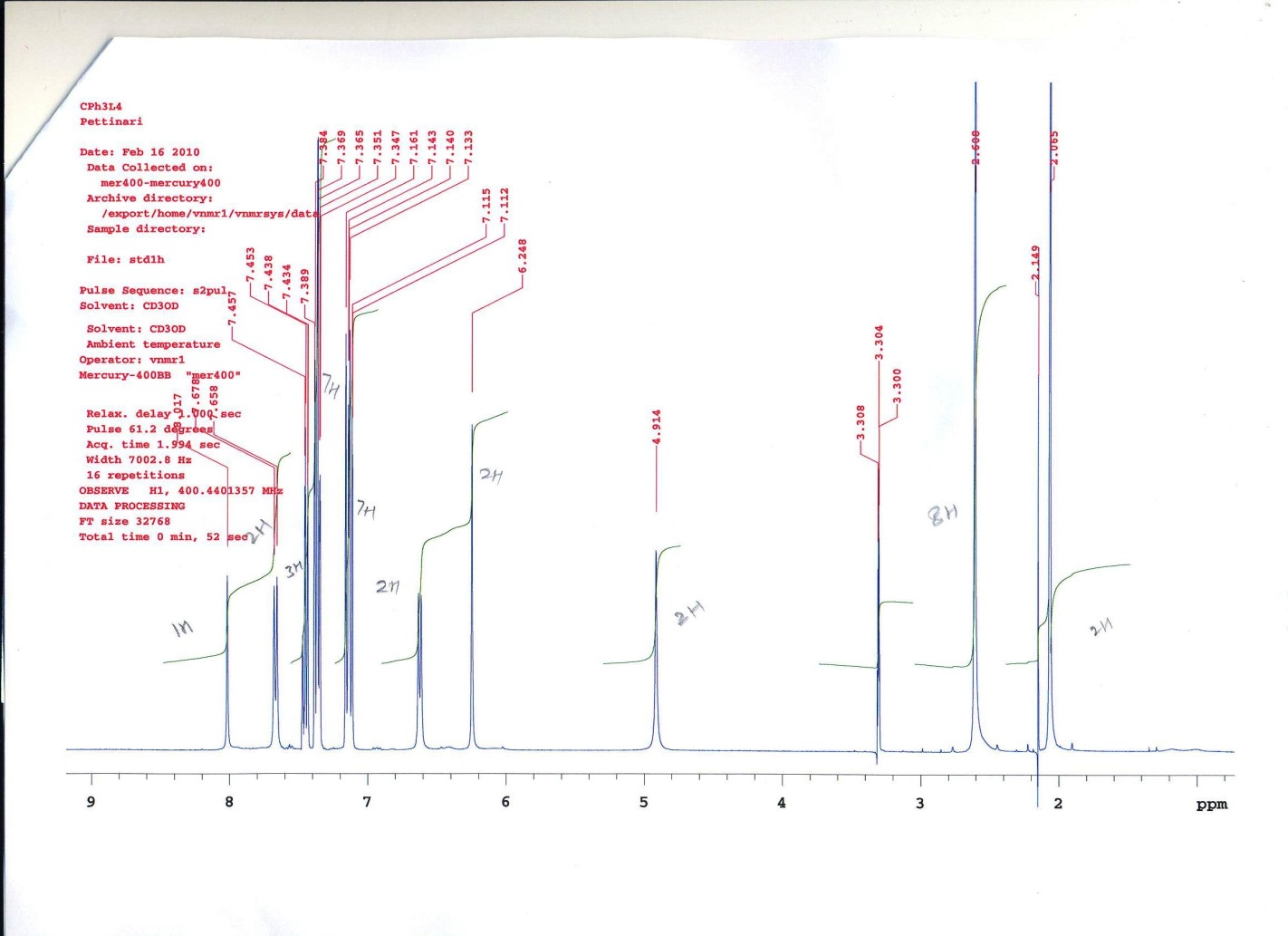


**Figures S5.** ^1^H NMR spectrum of complex **1.**


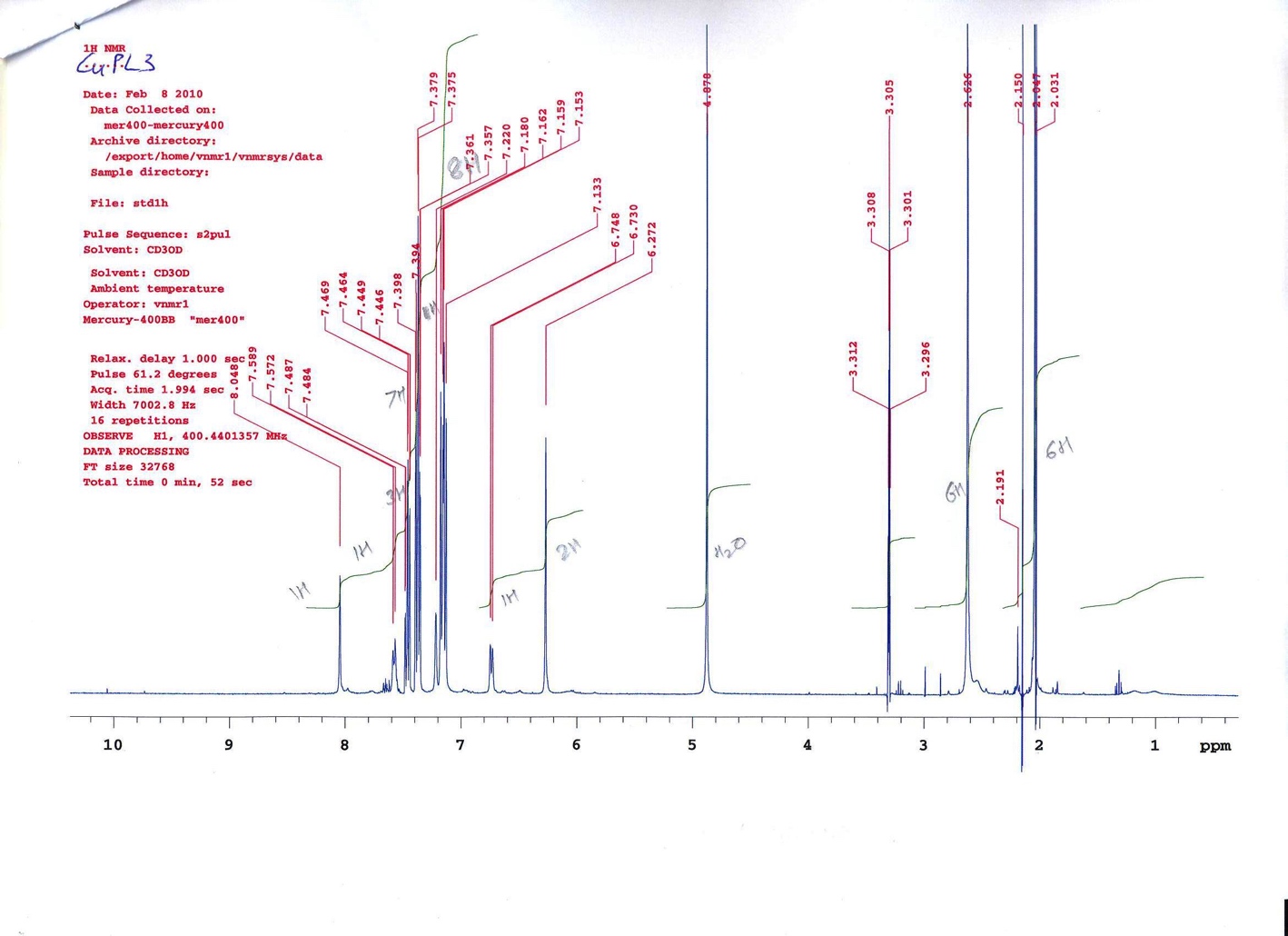


**Figures S6.** ^1^H NMR spectrum of complex **2.**


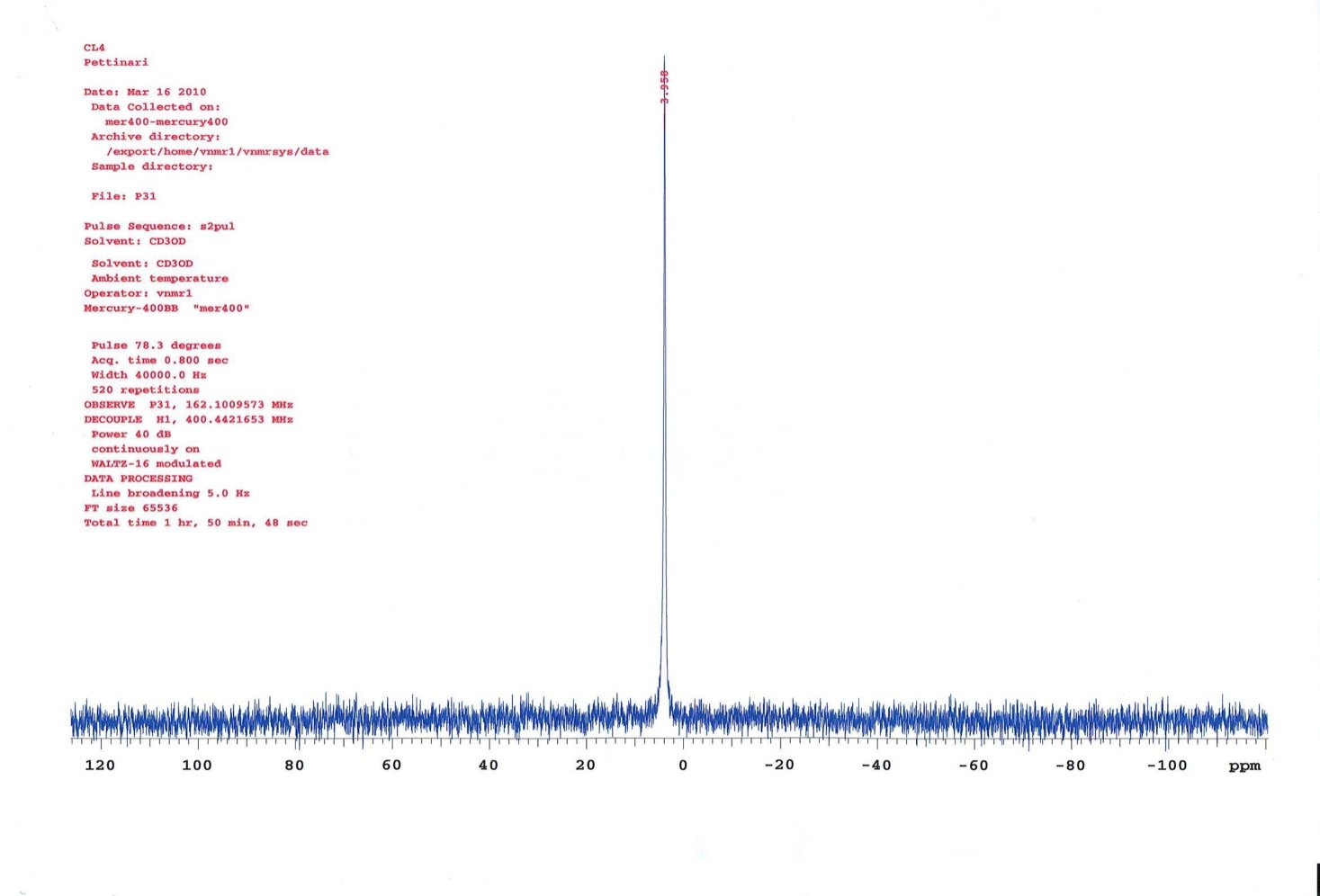


**Figures S7.** ^31^P NMR spectrum of complex **1.**


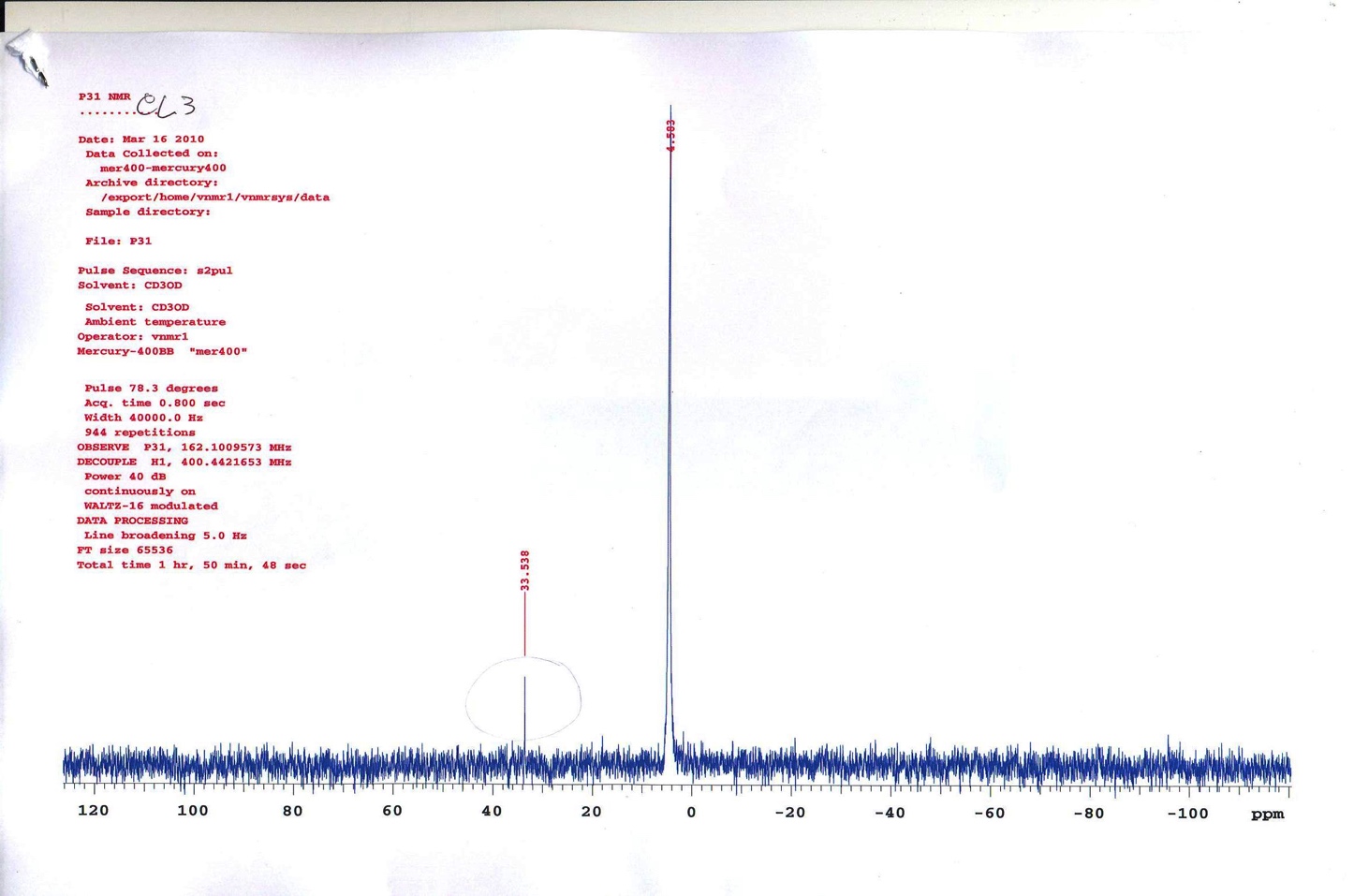


**Figures S8.** ^31^P NMR spectrum of complex **2.**

**1**

**2**

**Figure S9. Simulated UV-spectra of complexes 1 and 2**
